# LncRNA RPL34-AS1 suppresses the proliferation, migration and invasion of esophageal squamous cell carcinoma via targeting miR-575/ACAA2 axis

**DOI:** 10.1186/s12885-022-10104-6

**Published:** 2022-09-26

**Authors:** Hu Zhang, Enchun Pan, Ying Zhang, Chao Zhao, Qiwei Liu, Yuepu Pu, Lihong Yin

**Affiliations:** 1 Key Laboratory of Environmental Medicine Engineering, Ministry of Education, School of Public Health, Southeast University, 87 Dingjiaqiao Street, Nanjing, 210009 People’s Republic of China; 2 Huaian Center for Disease Control and Prevention, Huaian, 223001 People’s Republic of China

**Keywords:** Esophageal squamous cell carcinoma, lncRNA RPL34-AS1, miR-575, ACAA2

## Abstract

**Background:**

Long noncoding RNAs (lncRNAs) are abnormally expressed in a broad type of cancers and play significant roles that regulate tumor development and metastasis. However, the pathological roles of lncRNAs in esophageal squamous cell carcinoma (ESCC) remain largely unknown. Here we aimed to investigate the role and regulatory mechanism of the novel lncRNA RPL34-AS1 in the development and progression of ESCC.

**Methods:**

The expression level of RPL34-AS1 in ESCC tissues and cell lines was determined by RT-qPCR. Functional experiments in vitro and in vivo were employed to explore the effects of RPL34-AS1 on tumor growth in ESCC cells. Mechanistically, fluorescence in situ hybridization (FISH), bioinformatics analyses, luciferase reporter assay, RNA immunoprecipitation (RIP) assay and western blot assays were used to detect the regulatory relationship between RPL34-AS1, miR-575 and ACAA2.

**Results:**

RPL34-AS1 was significantly down-regulated in ESCC tissues and cells, which was negatively correlated with overall survival in ESCC patients. Functionally, upregulation of RPL34-AS1 dramatically suppressed ESCC cell proliferation, colony formation, invasion and migration in vitro, whereas knockdown of RPL34-AS1 elicited the opposite function. Consistently, overexpression of RPL34-AS1 inhibited tumor growth in vivo. Mechanistically, RPL34-AS1 acted as a competing endogenous RNA (ceRNA) of miR-575 to relieve the repressive effect of miR-575 on its target ACAA2, then suppressed the tumorigenesis of ESCC.

**Conclusions:**

Our results reveal a role for RPL34-AS1 in ESCC tumorigenesis and may provide a strategy for using RPL34-AS1 as a potential biomarker and an effect target for patients with ESCC.

**Supplementary Information:**

The online version contains supplementary material available at 10.1186/s12885-022-10104-6.

## Introduction

Esophageal carcinoma (EC), as one of current malignant digestive system cancers, causes the sixth mortality rate cancer in the worldwide and the fourth incident rate in China [1, 2]. According to the latest Global Cancer Statistics 2020, the number of EC cases were as high as 588,113 [1]. Esophageal squamous cell carcinoma (ESCC), the major type of EC in China accounting for approximately 90% of EC cases, remains one of the most lethal of malignancies and major health burden [3]. The prognosis of EC remains enormously poor, with a low overall five-year survival rate of about 20% [4, 5]. Therefore, it is necessary to provide novel insights into the underlying mechanisms of ESCC progression, which can identify potential therapeutic targets to formulate effective diagnosis as early as possible.

Studies have revealed that 98% of human genome transcripts RNAs are non-coding RNA (ncRNAs) with limited or no protein coding capabilities [6]. Long noncoding RNAs (lncRNAs), larger than 200 nucleotides, participate in almost all human biological processes, including transcriptional regulation, epigenetic regulation, cell differentiation, embryonic development, and key signaling of diseases [7, 8]. Abnormal expression of lncRNAs in various types of cancers illustrated clinical potential as biomarkers and therapeutic targets [9]. At present, studies on the occurrence and development of lncRNAs mediated regulation on ESCC progression are in full swing [10]. LncRNA PCAT6 promoted the progression of ESCC by suppressing the expression of genes associated with cell proliferation and migration [11]. EMT-related lncRNA DDX11-AS1 facilitated ESCC process through targeting miR-30d-5p to regulate SNAI1/ZEB2 expression and Wnt/beta-catenin pathway [12]. LncRNA LINC01554 acted as a promoter of ESCC through regulating G3BP2/HDGF axis to enhance ESCC cell migration and invasion [13]. In brief, these studies confirmed that lncRNAs served as clinical therapeutic targets for human ESCC.

Recent studies have found that lncRNAs played important functional roles in regulating the translation and decay of messenger RNA, relying on competitive endogenous RNAs (ceRNAs) to sequester microRNAs (miRNAs) from binding to their cognate mRNA targets [14, 15]. For instance, lncRNA NORAD contributed to CDDP resistance of ESCC by regulating miR-224-3p/MTDH axis and advancing nuclear accumulation of beta-catenin [16]. LncRNA SNHG8 facilitated cell proliferation and autophagy to upregulate ATG7 by sponging microRNA-588 in colorectal cancer (CRC) cells [17]. As a novel discovered lncRNA, RPL34-AS1 has attracted increasing attention and research [18]. RPL34-AS1 has been reported to exert a vital role in different types of cancers, including lung, cervical, papillary thyroid, colorectal and gastric cancers [19–23]. RPL34-AS1 suppressed growth and metastasis of EC through down-regulating RPL34 expression [24]. However, whether RPL34-AS1 participates in ESCC progression through the ceRNA network needs further exploration. In the present study, we comprehensively investigate the role of lncRNA RPL34-AS1 in the regulation of miR-575/ACAA2 signaling pathway in the carcinogenesis of ESCC. Collectively, our results provide the novel mechanism of RPL34-AS1 in ESCC progression, which may assist in the development of new therapeutic targets for ESCC intervention.

## Materials and methods

### Tissue samples

A number of 75 primary pairs of ESCC tissue samples and adjacent normal tissues (4–5 cm from tumor tissue) were obtained from Huai’an First People’s Hospital (Jiangsu, China) between June 2018 and June 2020. After segmented, the clinical samples were immediately put into liquid nitrogen and transferred to − 80 °C for conservation. Among them, there were 53 males and 22 females (age <  50 (*n* = 3), ≥50 (*n* = 72). The inclusion criteria were as follows: i) Han residents who have lived in Huai’an for more than 20 years; ii) Patients with esophageal cancer who have been identified as primary esophageal cancer by pathological examination or endoscopic diagnosis; iii) None of EC patients received any radiotherapy or chemotherapy treatments before surgery. This study was approved by the Southeast University Affiliated Zhongda Hospital Ethics Committee, and it was conducted in line with the Helsinki Declaration and written informed consents were obtained from all the participants of the study.

### Cell culture

The normal human esophageal squamous epithelial cell line (Het-1A), and ESCC cell lines (EC109, EC9706) were supplied by the Key Laboratory of the Environmental Medicine Engineering of Southeast University, Ministry of Education, China. EC109 and EC9706 cells were cultured in RPMI-1640 medium (Gibco, USA) and Het-1A was contained in DMEM medium (Gibco, USA), respectively. The cell culture medium complemented with 10% fetal bovine serum (FBS) (Biological Industries), 1% penicillin and streptomycin (Gibco, USA) in humidified air at 37 °C with 5% CO_2_.

### Reverse transcription-quantitative (RT-q) PCR

The total RNA was extracted using Trizol Reagent (Invitrogen, USA). The reverse transcription and cDNA amplification of qPCR reactions were conducted using PrimeScript™ RT reagent Kit and TB Green Premix Ex Taq II (Takara, Japan) with StepOnePlus system (Applied Biosystems, Carlsbad, CA, USA), respectively. β-actin and U6 were normalized as internal control. Bulge-loopTM miRNA qRT-PCR Primer Sets (one RT primer and a pair of qPCR primers for each set) specific for miR-575 and U6 were designed by RiboBio (Guangzhou, China). The relative quantification of lncRNA, miRNA and mRNA expression were analyzed applying the 2^-ΔΔCT^ method compared to internal control. The detailed sequences of primers were provided in Table S1.

### RNA fluorescent in situ hybridization (FISH)

To localize the cellular distribution of RPL34-AS1, the FISH assay was performed using the lncRNA FISH Probe and Fluorescent in Situ Hybridization Kit (Ribobio, China) according to the manufacturer’s guidelines. The probe cocktail included the 18S/cytoplasm probe, U6/nuclear probe, and lncRPL34-AS1 probe (DAPI and Cy3-labeled probes were synthesized for fluorescence signals). The results were captured by the OLYMPUS laser confocal microscope FV1000 (Olympus, Tokyo, Japan).

### Transcriptome sequencing (RNA-seq) analysis

EC109 cells transfected by RPL34-AS1 plasmid and scramble pcDNA were used for RNA-seq analysis. RNA quantification and quality assurance were evaluated by NanoDrop ND-1000. The mixed different sample libraries were sequenced by IlluminaNovaSeq6000 sequencer. Based on the correlation analysis and transcript expression level, the differentially expressed genes were analyzed. Accession ID for the RNA-seq data is GSE154450 (Gene Expression Omnibus, GEO).

### Bioinformatics analysis

The expression profiles of ESCC patients were retrieved from the Cancer Genome Atlas (TCGA) database. To perform the mechanisms underlying the role of RPL34-AS1 in ESCC, interaction and binding site between RNAs were identified by starBase v3.0 online database (http://starbase.sysu.edu.cn). The miRNA information in miRBase was used to perform target prediction based on the RPL34-AS1 sequence and the candidate miRNAs were screened by miRBase (http://www.mirbase.org) and TargetScan (http://www.targetscan.org/).

### Cell transfection

The small interfering RNAs (siRNAs) and plasmid were transfected into ESCC cells using StarFectII High-Efficiency Transfection Reagent (GenStar) in line with the manufacturer’s protocol. Three individual lncRPL34-AS1 siRNAs (si-RPL34-AS1 #1, #2, #3 and si-NC) and plasmid vector (pcDNA 3.1-RPL34-AS1 (pc-RPL34-AS1), pcDNA 3.1-NC (pc-NC)) and pcDNA 3.1-ACAA2 (pc-ACAA2) were purchased from KeyGEN BioTECH. The miR-NC, miR-575 mimics and miR-575 inhibitor were provided by Genomeditech. The all nucleotide sequences were listed in Table S2. After 48 h transfection, cells were acquired for RT-qPCR or western blot analysis.

### Cell proliferation assay

Cells were seeded into 96-well plates at a density of 8000 cells/well and incubated for 0, 24, 48 and 72 h. The Cell Counting Kit 8 (CCK-8, meilunbio) assay was used to detect cell viability and the 5-ethynyl-20-deoxyuridine (EdU) labeling/detection assay was applied to perform cell proliferation ability in accordance with the manufacturer’s instructions.

### Colony formation assay

After transfection for 48 h, the density of 1000 EC109 cells per well was seeded into a 6-well plate with a medium containing 10% FBS for 2 weeks. Finally, the cell colonies were immobilized with 4% paraformaldehyde for 30 min and dyed with 0.1% crystal violet for 20 min. The number of colonies were counted and analyzed using an inverted microscope.

### Transwell assay

Cell invasion assay was detected by a 24-well Mill cell chamber (corning 3422) using a Matrigel-coated membrane of 8 μm pore size (corning). After transfection, 2 × 10^5^ ESCC cells in 150 μl RPMI1640 medium without FBS were seeded in the upper chamber. The lower chamber was filled with 600 μl RPMI-1640 medium containing 10% FBS and incubated for 24 hours. The cells on the upper membrane surface were wiped with a cotton swab and cells invading to the lower chamber were fixed with 95% ethanol, stained with 0.1% crystal violet, dried, and counted under the microscope (original magnification, × 200) in five representative fields. The migration assay was conducted in a similar method without the Matrigel coating.

### Western blot assay

Cell protein was lysed in cold radioimmunoprecipitation assay buffer (RIPA buffer; Beyotime, Shanghai, China), and protein concentration was detected using a BCA Protein Assay Kit (Thermo Fisher Scientific). The extracted protein was separated by 10% SDS-PAGE and transferred onto polyvinylidene fluoride (PVDF, 0.45 μm) membranes (Immobilon®-P). After blocking with 5% skimmed milk powder for 1 h, the membranes were incubated with primary antibodies anti-ACAA2 (1:1000, Abcam), anti-β-actin (1:2000, Cell Signaling Technology) overnight at 4 °C. Then the secondary antibodies (anti-mouse/anti-rabbit) were applied to incubate membranes for 1 h at room temperature and the signals were detected by the SuperSignal West Femto Trial Kit (Thermo Fisher Scientific).

### Dual-luciferase reporter assay

The luciferase reporter vector PGL3-CMV-LUC-MCS (Genomeditech, Shanghai) was applied to design, integrate and insert into the sequences of RPL34-AS1 and ACAA2–3’UTR wild type (WT) and their corresponding mutant type (MuT) binding sites for miR-575. The fluorescence intensity was evaluated by the relative ratio of firefly luciferase (Luc) activity/Renilla luciferase (Rena) activity.

### RNA immunoprecipitation (RIP)

The EZ-Magna RIP Kit (EMD Millipore, Billerica, MA) was used to perform RNA immunoprecipitation (RIP) assay following the manufacturer’s protocol. The lysed cell was immunoprecipitated with anti-Argonaute 2 (AGO2) and anti-IgG antibody (EMD Millipore). Finally, the purified RNA was detected by RT-qPCR analysis.

### Tumor xenograft model

The BALB/c nude mice (male, 4-week-old) were obtained to perform the effect of RPL34-AS1 on tumor growth for tumor xenografts experiments (Jiangsu GemPharmatech, China). The ten BALB/c nude mice were randomly assigned into two groups. After transfected with overexpression RPL34-AS1 and control vector, the EC109 cells were subcutaneously injected into the right flank of the mice (5 × 10^6^, 200 μl) in tumor growth assay. The following formula: Volume = (length × width^2^) / 2 was applied to observe and calculate volumes of tumors every 3 days. After 2 weeks of the tumors generated, mice were euthanized with CO_2_. After examining the mice along with complete cardiac arrest and pupil dilation, the tumor weights, RT-qPCR and Hematoxylin and eosin (H&E) staining were used to detect subcutaneous tumor tissues. The antibodies against Ki-67 (Abcam, Cambridge, MA, USA) were detected for immunohistochemistry (IHC) assay. All animal procedures were performed in line with the National Institutes of Health Guide for the care and authorized by the Animal Care and Use Committee of Southeast University following the ARRIVE guideline.

### Statistical analysis

Data were analyzed using SPSS 23.0 (IBM, USA) and GraphPad Prism8.1 software. All results were expressed as the mean ± SD. The difference between two groups was analyzed using an unpaired two-tailed student’s t-test. One-way ANOVA followed by Dunnett’s post hoc test was used to analyze differences among more than two groups. The Kaplan-Meier method and log-rank test were applied to plot and estimate overall survival curves. A two-sided *p* value < 0.05 was identified as statistically significant.

## Results

### RPL34-AS1 was down-regulated in ESCC tissues and cells

**Fig. 1:**
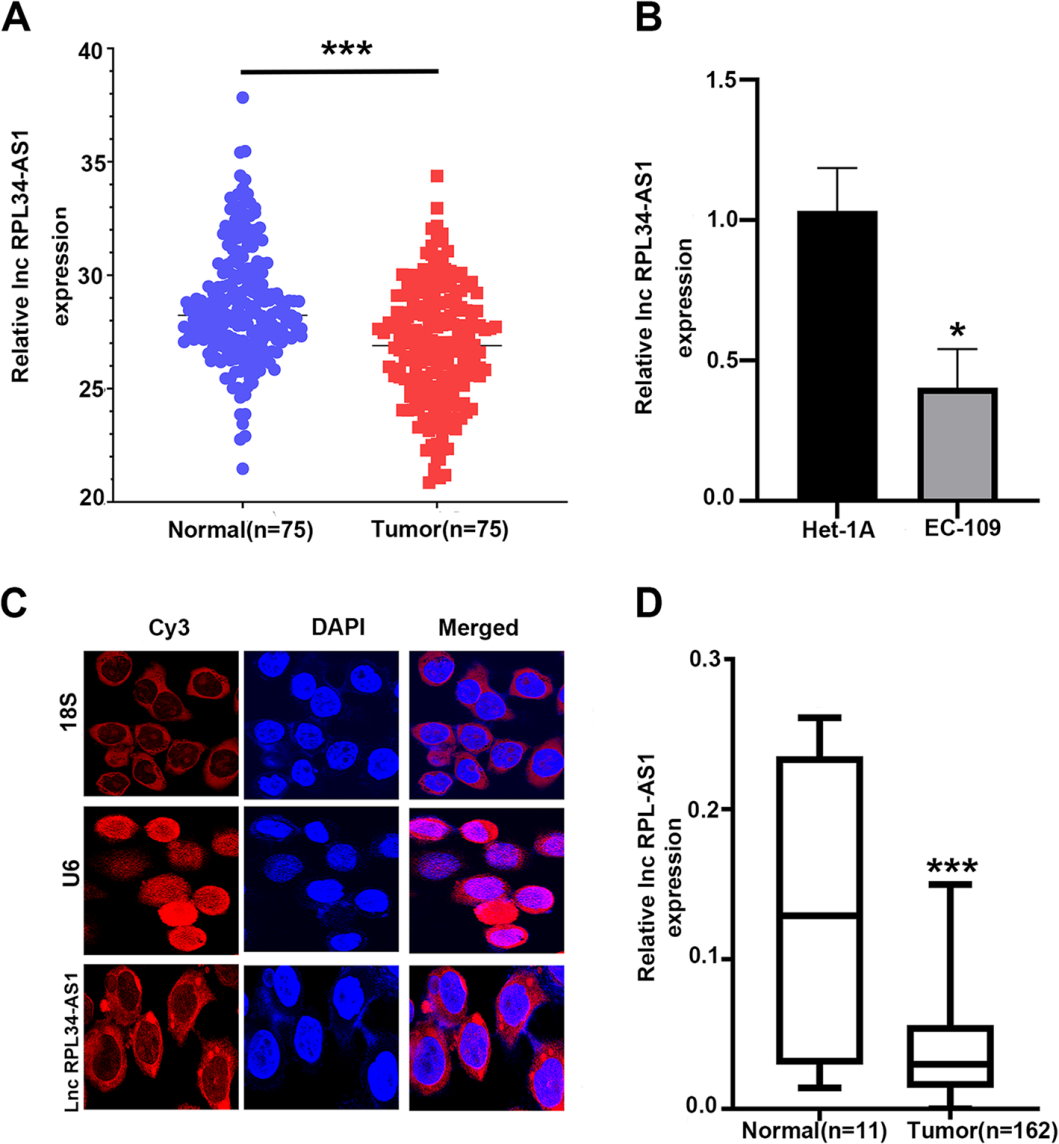
Identification and expression of RPL34-AS1 in ESCC tissues and cells. **A** Relative expression of RPL34-AS1 in ESCC and matched adjacent normal tissues was detected by RT-qPCR (*n* = 75). **B** Relative expression of RPL34-AS1 in cell lines was determined by RT-qPCR. **C** Using Single Molecule lncRNA Fluorescent in Situ Hybridization (lncRNA-FISH) to fix a position on RPL34-AS1. **D** RPL34-AS1 expression in ESCC tissues (*n* = 162) compared with noncancerous tissues (*n* = 11) analyzed using the TCGA database. Data were showed as mean ± SD. *P < 0.05, ***P < 0.001

To explore the role of RPL34-AS1 in ESCC, we first assessed RPL34-AS1 expression level in 75 paired primary ESCC and matched adjacent nontumor tissues by RT-qPCR. The results showed that the expression of RPL34-AS1 was significantly reduced in the ESCC tissue samples (Fig. 1A). Moreover, RPL34-AS1 expression was down-regulated in EC-109 cells compared with Het-1A cells (Fig. 1B). Furthermore, the results of FISH assay showed that the RPL34-AS1 transcripts were distributed mainly in the cytoplasm of EC109 cells (Fig. 1C), which indicated RPL34-AS1 might function in cytoplasm. To further investigate the expression pattern of RPL34-AS1 in ESCC, we also performed an analysis of RPL34-AS1 expression in a public microarray profile dataset from the Cancer Genome Atlas (TCGA) and used median RPL34-AS1 value as a cutoff, the results showed that RPL34-AS1 expression in 162 tumor samples of EC was significantly decreased, comparing with 11 adjacent tumor samples (Fig. 1D). The result of Kaplan-Meier survival analysis showed that ESCC patients with low RPL34-AS1 expression had a significant worse overall survival than those with high RPL34-AS1 expression (*p* = 0.018, Fig. S1). As shown in Table 1, the correlation analysis between RPL34-AS1 expression and clinicopathologic characteristics of these ESCC patients indicated that low expression of RPL34-AS1 was positively correlated with age (*p* = 0.008) and hard food (*p* = 0.042).

**Table 1:** Correlation between the clinicopathologic characteristics and RPL34-AS1 expression in ESCC

Characteristics	Class	*n* = 75	RPL34-AS1		*P*
			Low expression High expression		
Age (y)	< 50	3	0	3	0.008^**^
	≥50	72	59	13	
Sex	Male	53	40	13	
	Female	22	19	3	0.367
Tumor site	lower	17	11	6	0.671
	middle	50	43	7	
	upper	7	4	3	
Lymph node metastasis	no	52	43	9	0.078
	yes	23	16	7	
Smoking	light	28	24	4	0.158
	middle	9	5	4	
	deep	38	30	8	
Drinking	no	44	36	8	0.568
	yes	31	23	8	
Hot food	no	38	30	8	1.000
	yes	37	29	8	
Hard food	no	46	40	6	0.042^*^
	yes	29	19	10	
Fried food	no	53	39	14	0.496
	yes	13	11	2	
History of digestive disease	no	65	50	15	0.679
	yes	10	9	1	

* *P* < 0.05, **P < 0.01

### RPL34-AS1 suppressed ESCC cell proliferation, migration and invasion in vitro

**Fig. 2:**
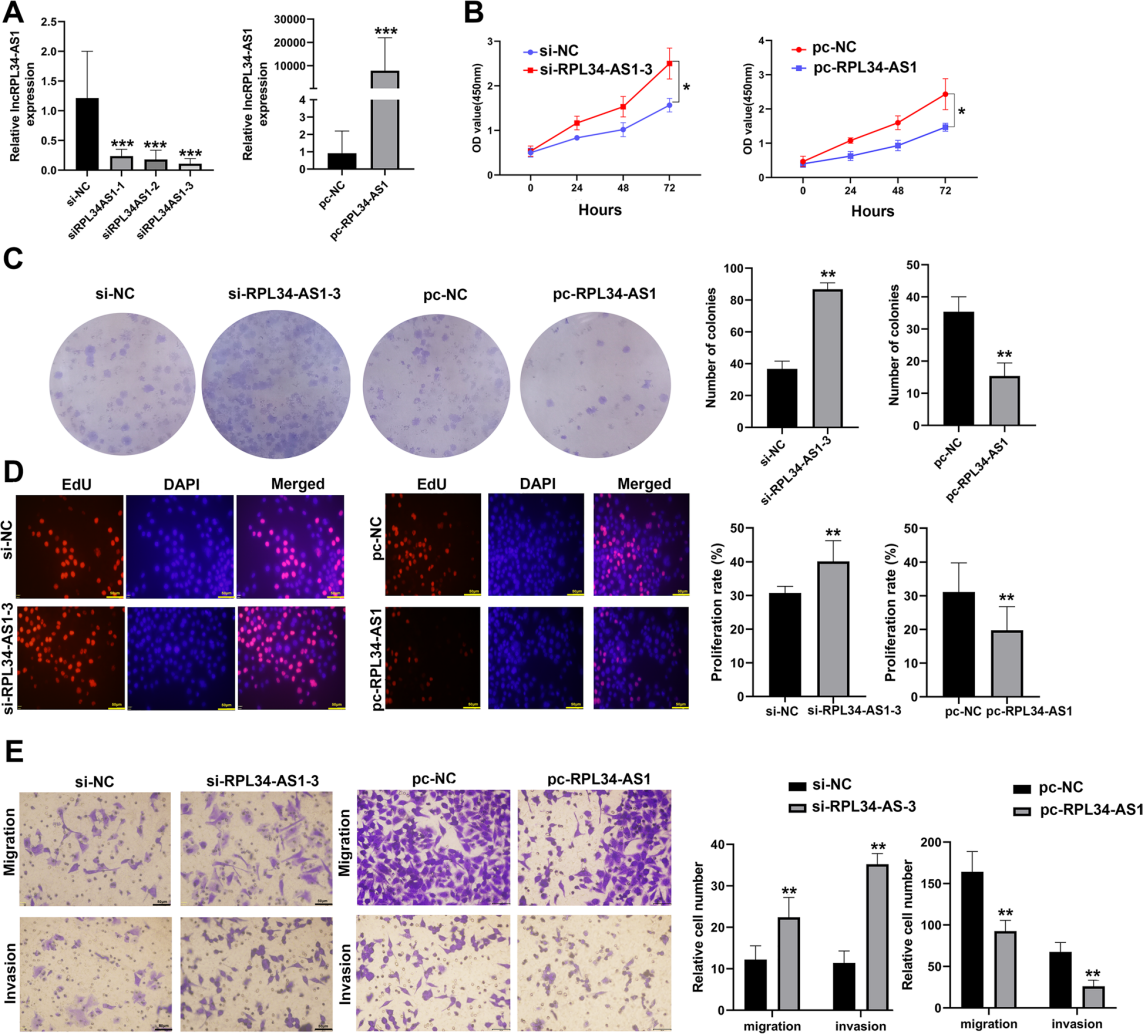
RPL34-AS1 suppressed ESCC cell growth and metastasis in vitro. **A** RPL34-AS1 expression level was detected in EC109 cells by RT–qPCR after up-regulated and down-regulated transfection. **B-D** CCK-8, colony formation and EdU assays were performed to determine the ability of proliferation in EC109 cells transfected with si-RPL34-AS1 or si-NC and transfected with pc-RPL34-AS1 or pc-NC. Scale bar, 50 μm. **E** Cell migratory and invasive capabilities were assessed by transwell assays in EC109 cells transfected with si-RPL34-AS1 or si-NC and transfected with pc-RPL34-AS1 or pc-NC. Scale bar, 50 μm. Data were showed as mean ± SD. *P < 0.05, **P < 0.01, ***P < 0.001

Given that RPL34-AS1 was down-regulated in ESCC, loss- and gain-of-function approaches transfected with pc-RPL34-AS1 or si-RPL34-AS1 were employed to determine the biological function of RPL34-AS1 in ESCC cells. The RT-qPCR analysis confirmed that RPL34-AS1 expression was successfully down-regulated or up-regulated in EC109 cells (Fig. 2A). CCK-8 assays confirmed that RPL34-AS1 downregulation significantly reinforced the proliferation viability, whereas the RPL34-AS1 upregulation performed opposite effects (Fig. 2B). Colony formation assays demonstrated that the downregulation of RPL34-AS1 obviously promoted cloning capabilities of EC109 cells and markedly reduced by the overexpression of RPL34-AS1 (Fig. 2C). Similarly, EdU assays further revealed that knockdown of RP34-AS1 greatly increased the percentages of EdU-positive cells, which considerably decreased at upregulation of RPL34-AS1 (Fig. 2D). Moreover, transwell assays were executed to examine the effects of RPL34-AS1 on invasion and migration of EC109 cells. The results showed that the capabilities of migratory and invasive EC109 cells were remarkably enhanced by downregulation of RPL34-AS1 but significantly suppressed by overexpression of RPL34-AS1 (Fig. 2E). These assays suggested that RPL34-AS1 inhibited invasion and migration of EC109 cells.

### RPL34-AS1 was a sponge for miR-575

**Fig. 3:**
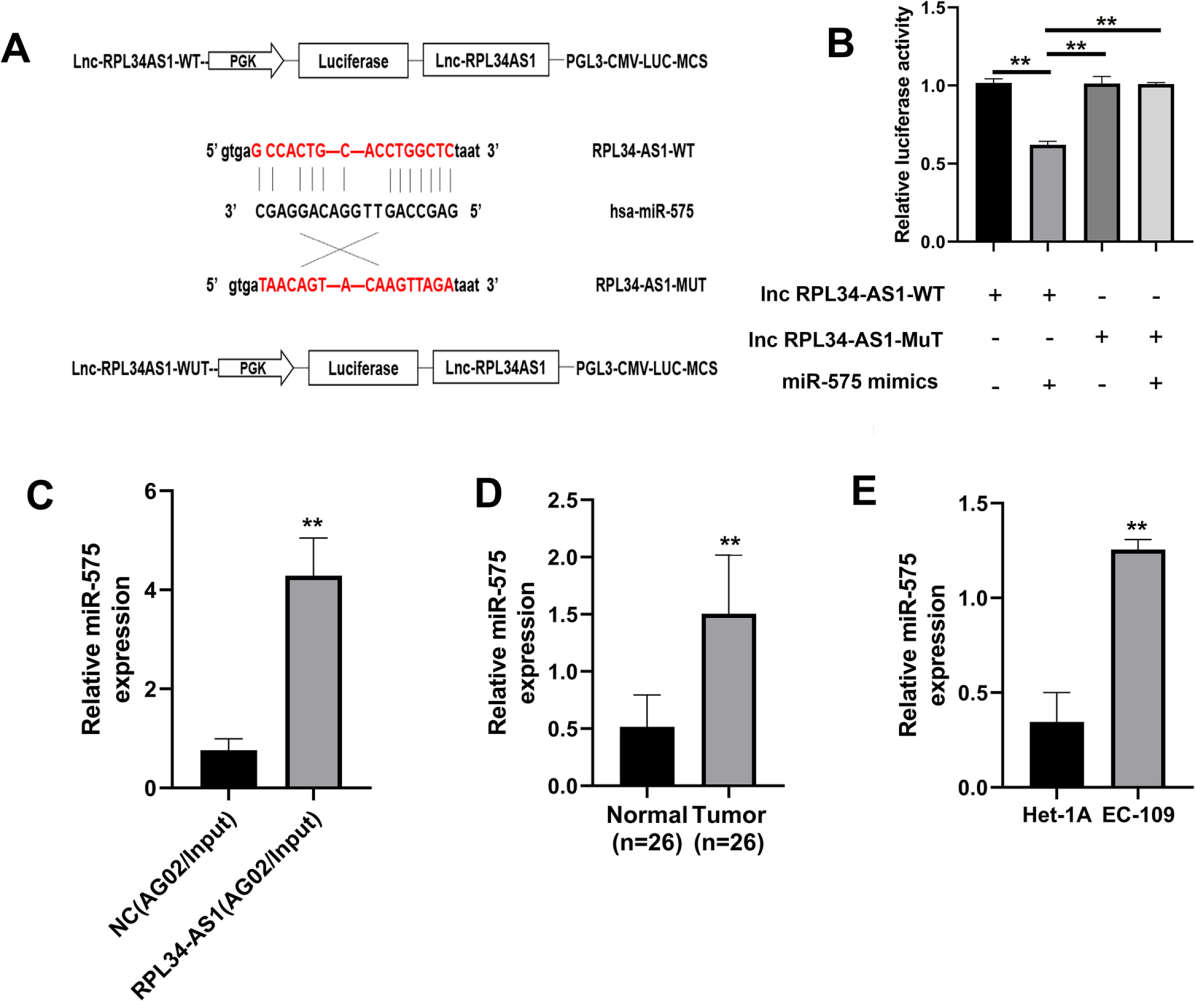
RPL34-AS1 was a sponge for miR-575. **A** Prediction for miR-575 binding elements on RPL34-AS1. **B** Mutations were generated in lncRPL34-AS1 binding sites of miR575. Luciferase activity in EC109 cells co-transfected with miR-575 mimics/miR-NC and WT/MuT lncRPL34-AS1. Data were presented as the relative ratio of firefly luciferase activity to Renilla luciferase activity. **C** The anti-AGO2 RIP immunoprecipitations were measured by RT-qPCR. **D**, **E** The expression of miR-575 was performed by RT-qPCR in ESCC tissues and cells. Data were showed as mean ± SD. *P < 0.05, **P < 0.01

The prediction results of miRanda and TargetScan displayed the ternary relationship of lncRNA-miRNA in the form of a network diagram (Fig. S2). Our results revealed that miR-575 had the highest score. The binding sites of RPL34-AS1 and miR-575 was discovered (Fig. 3A). Correspondingly, we then exemplified the binding relationship between RPL34-AS1 and miR-575. The results showed that the luciferase activity of RPL34-AS1 WT reporter vector was significantly reduced by miR-575 mimics, compared with the empty vector and mutant reporter vector (Fig. 3B). Followed by an anti-AGO2 RIP assay was implemented to validate the binding relationship between RPL34-AS1 and miR-575, the results of RIP implied that the expression of miR-575 in the RPL34-AS1 overexpression group was specifically higher than the NC group (Fig. 3C). Next, the results of RT-qPCR indicated significant upregulation of miR-575 in ESCC tissues relative to adjacent normal tissues and significant overexpression of miR-575 in EC-109 cells relative to Het-1A cells (Fig. 3D and E).

### RPL34-AS1 regulated ACAA2 expression through miR-575

**Fig. 4:**
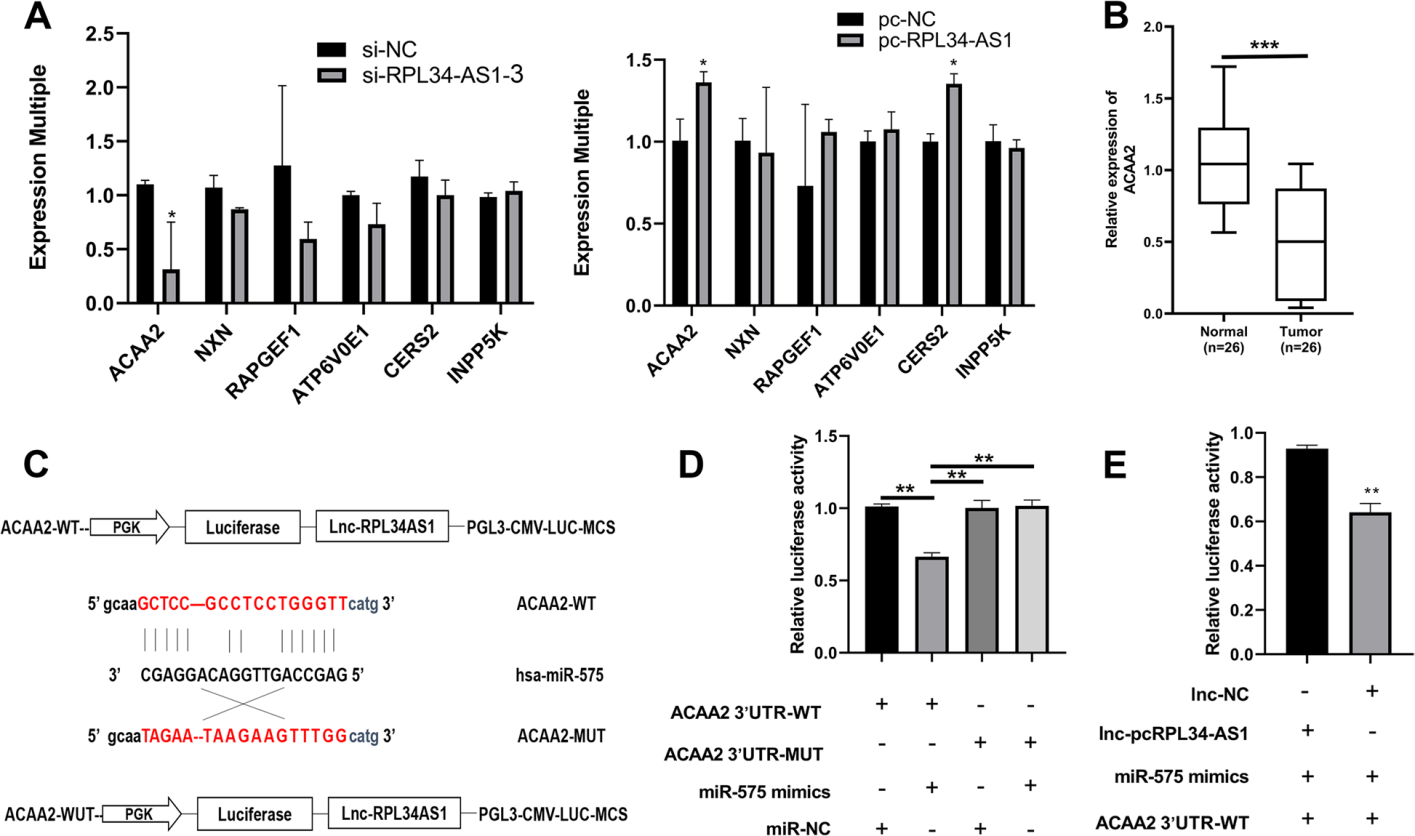
RPL34-AS1 regulated ACAA2 expression through miR-575. **A** The targeted genes expression was detected by RT-qPCR after knockdown or overexpression of lncRPL34-AS1 in EC109 cells. **B** The expression of ACAA2 was performed by RT-qPCR in ESCC tissues. **C** Prediction for miR-575 binding elements on ACAA2. **D** Mutations were generated in ACAA2 3′-UTR binding sites of miR-575. DLR analysis was performed to confirm miR-575 shares binding sites with ACAA2. The luciferase activity was detected in cells co-transfected with miR-575 mimics/miR-NC and wild-type/mutant ACAA2; and **E** to further evaluate the relationship of RPL34-AS1, miR-575, and ACAA2 by use of DLR analysis. Data were showed as mean ± SD. *P < 0.05, **P < 0.01, ***P < 0.001

In order to further investigate the downstream gene expression of RPL34-AS1 through miR-575, we examined the mRNA expression profiles in EC109 cells after overexpression of RPL34-AS1. As shown in Fig. S3, we used differentially expressed genes of RNA-seq results and ceRNA targets to take the intersection in Venny and DAVID bioinformatic analysis. Herein 6 targeted genes were selected for subsequent mechanism research in line with fold change and *p* value. The result of ACAA2 expression was proved to show consistent trend with RPL34-AS1 after knockdown and overexpression of RPL34-AS1 in the EC109 and EC9706 cells (Fig. 4A, Fig. S4). Results of RT-qPCR indicated significant downregulation of ACAA2 in ESCC tissues relative to adjacent normal tissues (Fig. 4B). The binding sites of ACAA2 and miR-575 was found (Fig. 4C). To decipher the regulatory mechanisms of miR-575 on ACAA2, we transfected luciferase reporter vector harboring 3′ UTR (WT and MuT) of ACAA2 into EC109 cells and luciferase activity was then evaluated in the transfection of miR-575 mimics. As compared to the control vector, miR-575 mimics significantly reduced the luciferase activity of the ACAA2 reporter vector (ACAA2 3′ UTR-WT) (Fig. 4D). Furthermore, to confirm the role of RPL34-AS1 on regulation of miR-575/ACAA2, we then set up another dual-luciferase (DLR) analysis and divided into two groups: Group 1 (RPL34-AS1 + ACAA2 WT + miR-575 mimics) and Group 2 (NC + ACAA2 WT + miR-575 mimics), the fluorescence intensity in Group 2 was reduced by 31% compared with Group 1 (Fig. 4E). These results enlightened that RPL34-AS1 may regulate ACAA2 expression by competitively interacting with miR-575.

### MiR-575 promoted ESCC cells proliferation, migration and invasion in vitro by targeting ACAA2

**Fig. 5:**
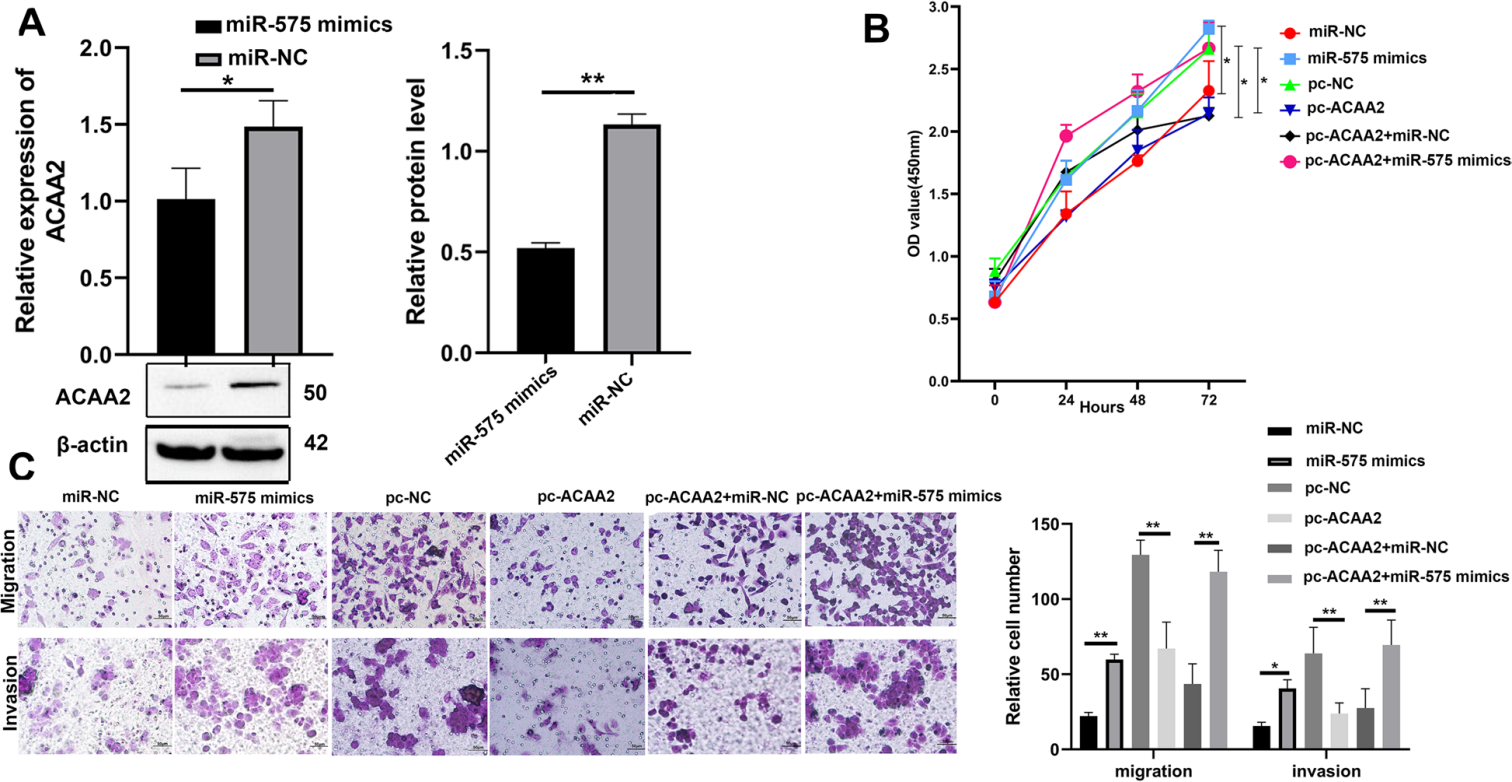
MiR-575 promoted ESCC cells proliferation, migration and invasion in vitro by targeting ACAA2. **A** Relative mRNA expression and protein level of ACAA2 were evaluated by RT-qPCR and western blot analysis in EC109 cells transfected with the miR-575 mimics. **B** CCK-8 assays were performed to determine the ability of proliferation in EC109 cells transfected with miR-575 mimics, miR-NC, pc-ACAA2, pc-NC, pcACAA2 + miR-NC and pcACAA2 + miR-575 mimics. **C** The cell migratory and invasive capabilities were assessed by transwell assays in EC109 cells transfected with miR-575 mimics, miR-NC, pc-ACAA2, pc-NC, pcACAA2 + miR-NC and pcACAA2 + miR-575 mimics. Scale bar, 50 μm. Data were showed as mean ± SD. *P < 0.05, **P < 0.01

As few studies had investigated the role of miR-575 in ESCC, we performed the mechanistical function of miR-575 by targeting ACAA2. The results of RT-qPCR and western blot displayed that substantially decreased expression of ACAA2 in miR-575 mimics group in the EC109 and EC9706 cells (Fig. 5A, Fig. S5A). The upregulation of miR-575 significantly enhanced the proliferation viability, migratory and invasive capabilities of EC109 and EC9706 cells. Furthermore, the overexpression of ACAA2 (pc-ACAA2) repressed the proliferation viability, migratory and invasive capabilities of EC109 and EC9706 cells, whereas the above effects were reversed by miR-575 mimics (Fig. 5B, C, Fig. S5B, C).

### RPL34-AS1 suppressed ESCC cell growth and metastasis through RPL34-AS1/miR-575/ACAA2 axis

**Fig. 6:**
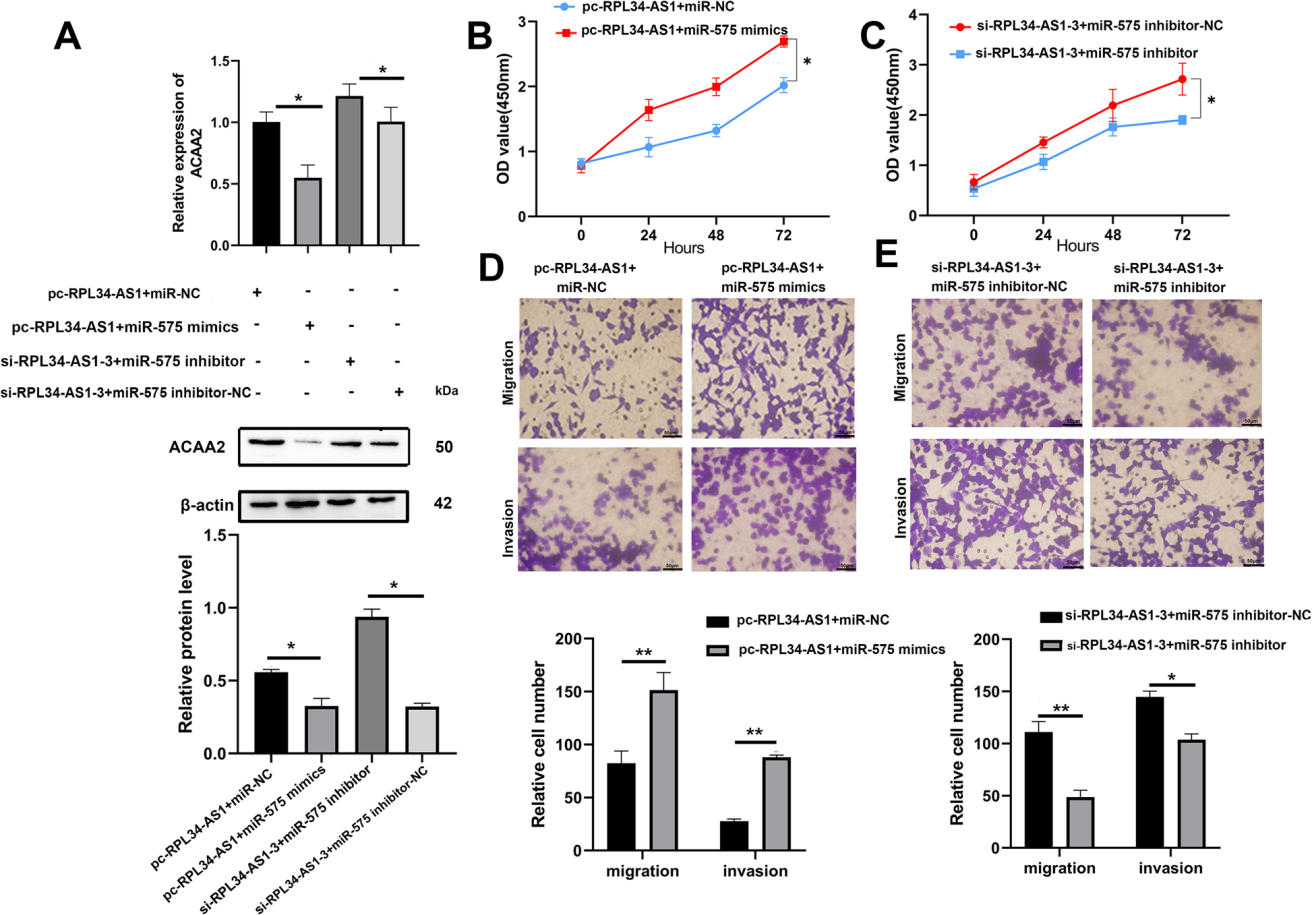
RPL34-AS1 suppressed ESCC cell growth and metastasis through lncRPL34-AS1/miR-575/ACAA2 axis. **A** Relative mRNA and protein level of ACAA2 were evaluated by RT-qPCR and western blot in EC109 cells transfected with indicated miR-575 mimics, inhibitor, miR-NC, si-RPL34-AS1 or pc-RPL34-AS1, respectively. **B-E** CCK-8 assays were performed to determine the ability of proliferation and cell migratory and invasive capabilities were assessed by transwell assays in EC109 cells transfected with indicated miR-575 mimics, inhibitor, miR-NC, si-RPL34-AS1 or pc-RPL34-AS1, respectively. Scale bar, 50 μm. Data were showed as mean ± SD. *P < 0.05, **P < 0.01

Accordingly, to confirm whether RPL34-AS1 served its suppressor function through RPL34-AS1/miR-575/ACAA2 axis, rescue experiments were enforced using inhibitors and mimics. The gene expression and protein level of ACAA2 induced by knockdown or overexpressing RPL34-AS1 were inverted by miR-575 inhibitor or mimics, respectively (Fig. 6A). We attempted to perform whether the biological function of RPL34-AS1 in EC109 cells could be reversed by miR-575 inhibitor or mimics. The results demonstrated that the miR-575 mimics reversed the proliferation, invasion and migration inhibiting effects leaded by overexpression of RPL34-AS1 in EC109 cells, nevertheless miR-575 inhibitor counteracted the promoting effects induced by knockdown of RPL34-AS1 in EC109 cells (Fig. 6B-E). In summary, these data strongly suggested that RPL34-AS1 suppressed ESCC cell growth and metastasis through RPL34-AS1/miR-575/ACAA2 axis.

### Overexpression of RPL34-AS1 restrained tumorigenesis and growth of ESCC in vivo

**Fig. 7:**
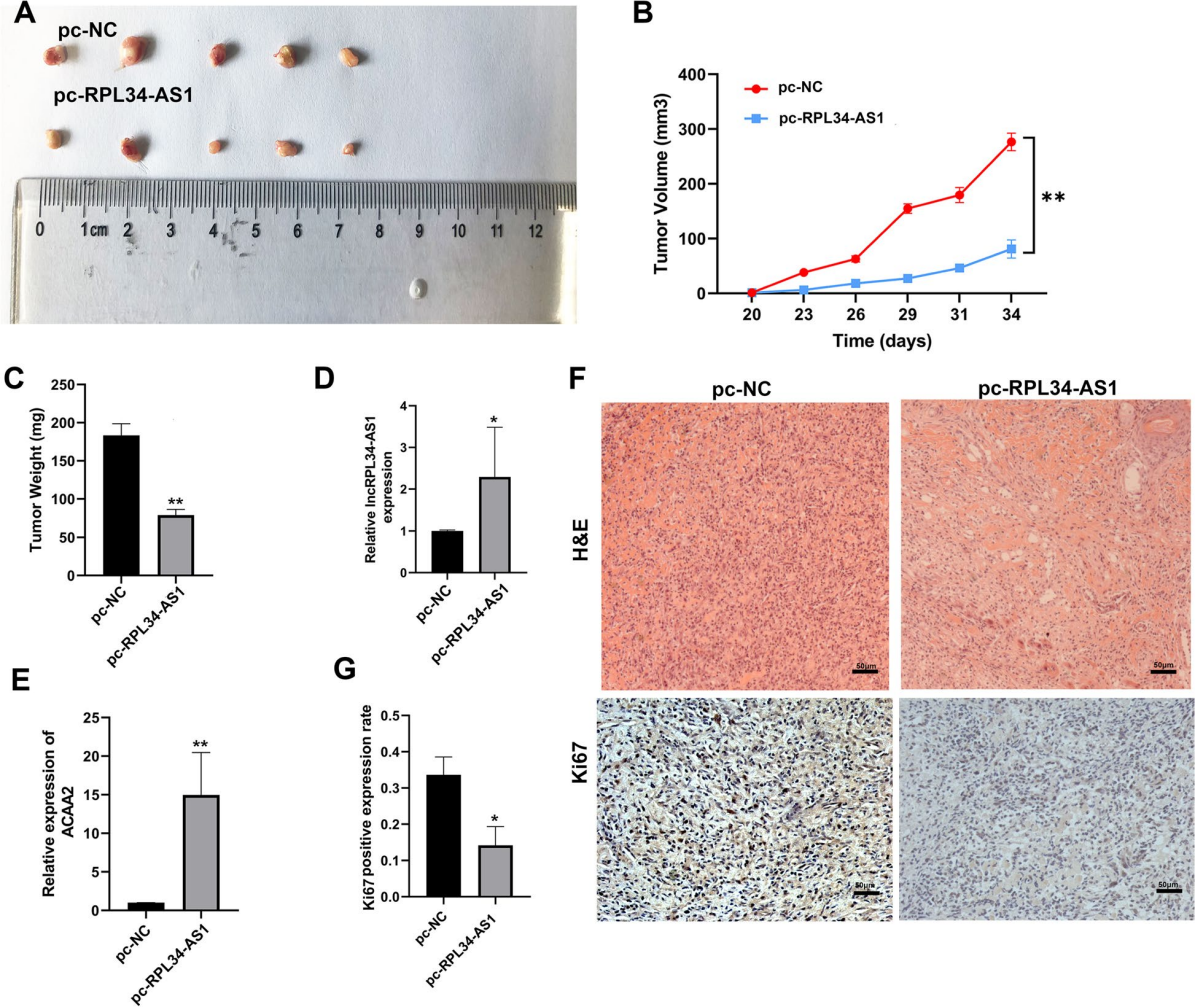
Overexpression of RPL34-AS1 restrained tumorigenesis and growth of ESCC in vivo. **A** Image of subcutaneous tumor tissues in RPL34-AS1-overexpressing group and control group. **B** Analysis of tumor volume of mice measured every three days. **C** The relative weights of tumors were evaluated. **D**, **E** Relative expression levels of RPL34-AS1 and ACAA2 were observed in subcutaneous tumor tissues by RT-qPCR. **F** The xenografts were H&E stained and expression of Ki67 was measured by immunohistochemistry. Scale bar, 50 μm. **G** The result of Ki67 staining cell positive expression. Data were showed as mean ± SD. *P < 0.05, **P < 0.01

To further determine the function of RPL34-AS1 on tumor growth in vivo, The BALB/c nude mice were injected by EC109 cells transfected with pc-RPL34-AS1 or control vector. 34 days after injection, the tumors were collected. The tumors formed in the pc-RPL34-AS1 group were substantially smaller than those in the control group (Fig. 7A). The results of tumor growth curves and weights showed that RPL34-AS1 overexpression obviously decreased tumor growth in mice (Fig. 7B, C). Tumor tissues were collected for RT-qPCR analysis of RPL34-AS1 and ACAA2. Compared with the control group, the higher expression of RPL34-AS1 and ACAA2 were detected in tumor tissues acquiring from RPL34-AS1 overexpression group (Fig. 7D, E). Furthermore, H&E and IHC for Ki-67 were performed to detect the expression of Ki-67, and results showed that RPL34-AS1 overexpression caused decreased Ki-67 expression (Fig. 7F). The result of Ki-67 staining cell positive expression showed that RPL34-AS1 overexpression caused lower Ki-67 cell positive expression percentage (Fig. 7G). Altogether, these results revealed the suppressive role of RPL34-AS1 in ESCC progression.

## Discussion

LncRNAs are encoded by a less explored region of the human genome and may retain impairing cancer drivers. Recently, they have attracted attention as potential key layers of cancer cell regulation [8, 25]. Meanwhile, increasing studies have revealed that lncRNAs exerted biological and clinical relevance of proliferation and metastasis-associated function in ESCC [26–28]. In the present study, we found that RPL34-AS1 was obviously downregulated in ESCC cells and tissues, and low RPL34-AS1 expression was associated with poor prognosis of EC patients. Next, loss- and gain-of-function assays indicated that RPL34-AS1 acted as a tumor suppressor in vivo and vitro of ESCC. Furthermore, given the cellular distribution of RPL34-AS1 mainly in cytoplasm, we utilized ceRNA regulatory network to explore the underlying mechanisms of RPL34-AS1.

Increasing evidence has suggested that abnormal expression of lncRNAs function acts as tumor suppressors or promoters [29]. Previous studies have reported the carcinogenesis of RPL34-AS1. For instance, downregulation of RPL34-AS1 restrained glioma cell proliferation by inhibiting angiogenesis through decreased ERK/AKT signaling [18]. RPL34-AS1-regulated RPL34 suppressed cervical cancer proliferation, invasion, and metastasis through modulation of the MDM2-P53 signaling pathway [20]. Overexpression of RPL34-AS1 inhibited colorectal cancer cell proliferation, invasion, and apoptosis, in which RPL34-AS1 may play a regulatory role through the RPL34-AS1/miR-93/PTEN axis [30]. Validation of paired gastric cancer specimens found that decreased expression of RPL34-AS1 was normally correlated with larger cancer tissue sizes [23]. Also, RPL34-AS1 acted a suppressor of thyroid papillary carcinoma via competitively binding miR-3663-3p/RGS4 axis [21]. This was consistent with the results of the present study, suggesting that RPL34-AS1 may act as a suppressor in the occurrence and progression of ESCC.

It is emerging that one of the most popular functional models by which lncRNAs regulate gene expression is to interact with miRNAs as ceRNAs that bind to miRNA response elements (MREs) and repress targeting mRNAs [31, 32]. Here, analyzed by bioinformatics analysis, RPL34-AS1 was predicted to harbor miRNA-binding sites of miR-575 in the RPL34-AS1 sequence, which was further confirmed by luciferase reporter assay and RIP assay. As important roles in several kinds of diseases, miR-575 has attracted more and more attention [33–35]. MiR-575 increased proliferation and inhibited apoptotic death of gastric cancer cells by regulating PTEN both in vitro and vivo [36]. Knockdown of miR-575 significantly suppressed proliferation and invasion of gallbladder cancer (GBC) cells via targeting p27 Kip1 [37]. Overexpression of lncRNA MIR31HG decreased the expression of miR-575 by targeting ST7L, which acted as a tumor suppressor in hepatocellular carcinoma [38]. In our study, the expression of RPL34-AS1 was negatively associated with miR-575, and a significant reciprocal repression feedback loop present in ESCC cells. Importantly, our results showed that miR-575 acted as a tumor promoter in ESCC, and subsequent rescue experiments further confirmed that miR-575 mimics or inhibitor reversed the tumor suppressor roles of RPL34-AS1. Together, our results revealed that RPL34-AS1 exerted an inhibitory role in the progression of ESCC by sponging miR-575.

Furthermore, miRNAs could function in multiple ways, including by targeting the 3′-UTR of target genes to regulate lncRNAs [39]. The current study predicted that the 3′-UTR of ACAA2 contained complementary sites to miR-575, suggesting that ACAA2 may be a target gene of miR-575. Acetyl CoA acyltransferase 2 (ACAA2) is a key enzyme in the fatty acid oxidation pathway which catalyzes the last step of mitochondrial beta-oxidation and participates in various pathways related to lipid metabolism [40]. Low expression of ACAA2 was correlated with low overall survival rates in patients with cancer, including breast cancer, ovarian cancer and lung cancer and acted as a tumor suppressor [41–43]. To date, whether and how lncRNA contributed to ACAA2-induced progression in ESCC remains elusive. In the present study, our results indicated that RPL34-AS1 interacted with miR-575 to promote the expression of ACAA2, and inferred a novel mechanistic role of RPL34-AS1/miR-575/ACAA2 axis in regulating the progression of ESCC. The upregulation of miR-193a-5p inhibited 3 T3-L1 preadipocyte differentiation, leading to a decrease in fatty acid related gene ACAA2 [44]. Furthermore, nonalcoholic steatohepatitis (NASH) related differential lncRNAs were associated with predicted protein-coding targets of ACAA2 [45]. In addition, we revealed that ACAA2 was downregulated in ESCC and ACAA2 overexpression clearly inhibited the proliferation, migration and invasion of ESCC cells. Hence, we further demonstrated the suppressive role of ACAA2 and provided evidence for the posttranscriptional regulation of ACAA2 by RPL34-AS1 in ESCC.

In our study, we investigated ceRNA regulating networks of RPL34-AS1 involving the downstream gene in the progression of ESCC. However, RPL34-AS1 regulated the development of ESCC through other mechanisms such as RNA-binding protein, post-transcriptional regulation and signaling pathway feedback required further investigation. Therefore, a deeper understanding the therapeutic potential of RPL34-AS1 in ESCC which warrants additional detailed studies.

## Conclusion

We identified the novel lncRNA RPL34-AS1 was obviously downregulated in ESCC and functioned as a tumor suppressor in vitro and vivo. Furthermore, we demonstrated that RPL34-AS1 acted as an endogenous sponge of miR-575 and subsequently promoted ACAA2 expression to inhibit ESCC cells proliferation, invasion and migration, suggesting that RPL34-AS1 may be used as a therapeutic target for patients with ESCC.

## Supplementary Information

**Additional file 1: Supplementary Table S1.** Primers for RT-qPCR. **Supplementary Table S2.** Nucleotide sequences for transfection. **Supplementary Fig. S1.** Kaplan-Meier survival curves of ESCC patients with low and high RPL34-AS1 expression. **Supplementary Fig. S2.** The lncRPL34-AS1-miRNA-mRNA network diagram was screened by miRanda and TargetScan algorithm. **Supplementary Fig. S3.** The RNA-seq results and ceRNA targets to take the intersection in Venny and DAVID bioinformatic analysis. **Fig. S3.** Venny and DAVID bioinformatic analysis. A. The intersection mRNA genes of RNA-seq expressed genes and ceRNA targets were displayed in Venny. B. The functional pathways were enriched via DAVID bioinformatics resources. **Supplementary Fig. S4.** The targeted genes expression after knockdown or overexpression of lncRPL34-AS1 in EC9706 cells. **Fig. S4.** The targeted mRNAs expression was detected by RT-qPCR. A-B. Downregulation and of upregulation of RPL34-AS1 in EC9706 cells. Data were showed as mean ± SD. *P < 0.05, **P < 0.01. **Supplementary Fig. S5.** MiR-575 promoted ESCC cells proliferation, migration and invasion in vitro by targeting ACAA2 in EC9706 cells. **Fig. S5.** MiR-575 acted a promoter of EC9706 cells proliferation, migration and invasion in vitro by targeting ACAA2. A. Relative mRNA expression and protein level of ACAA2 were evaluated by RT-qPCR and western blot analysis in EC9706 cells transfected with the miR-575 mimics. B. CCK-8 assays were performed to determine the ability of proliferation in EC9706 cells transfected with miR-575 mimics, miR-NC, pc-ACAA2, pc-NC, pcACAA2 + miR-NC and pcACAA2 + miR-575 mimics. C. The cell migratory and invasive capabilities were assessed by transwell assays in EC9706 cells transfected with miR-575 mimics, miR-NC, pc-ACAA2, pc-NC, pcACAA2 + miR-NC and pcACAA2 + miR-575 mimics. Scale bar, 50 μm. Data were showed as mean ± SD. *P < 0.05, **P < 0.01. **Supplementary Fig. S6.** The original blots of images of Fig. 5A. **Supplementary Fig. S7.** The original blots of images of Fig. 6A. **Supplementary Fig. S8.** The original blots of images of Fig. S5A.

## Data Availability

The datasets generated and/or analysed during the current study are available in the Gene Expression Omnibus (GEO) datasets (https://www.ncbi.nlm.nih.gov/gds), and the accession number was GSE154450.

## Abbreviations

ESCC: Esophageal squamous cell carcinoma
EC: Esophageal cancer
LncRNA: Long nocoding RNA
ceRNA: Competing endogenous RNA
miRNA: MicroRNA
mRNA: Messenger-RNA
RIP: RNA immunoprecipitation
ACAA2: Acetyl CoA acyltransferase 2
