# Supplementary material for: LncRNA RPL34-AS1 suppresses the proliferation, migration and invasion of esophageal squamous cell carcinoma via targeting miR-575/ACAA2 axis
Source: BMC Cancer. 2022 Sep 26;22:1017. doi: 10.1186/s12885-022-10104-6 (PMC9511711; doi:10.1186/s12885-022-10104-6)

**Supplementary Table S1. Primers for RT-qPCR.**

| Gene name |  | Primer sequences (5’ to 3’) |
| --- | --- | --- |
| RPL34-AS1 | Forward | TACAGGCACTCGCCATCAT |
|  | Reverse | CCATTTATAGCACCTAGAACAGC |
| ACAA2 | Forward | TATTGGTCCTGTCCCTGCTAT |
|  | Reverse | CTCCTCCATTCACATTGGTTTTA |
| NXN | Forward | GTGGAGGTGCTGAACGACG |
|  | Reverse | CGGCTACGAAGAACAGAAGG |
| CERS2 | Forward | CTGATGTCAAGCGAAAGGATT |
|  | Reverse | ACCAGTCGGGTGATGATAAAA |
| INPP5K | Forward | CAGAATTGTGAGGGGCGAGAC |
|  | Reverse | TGTAGGTGGGCGGGAAGAGTA |
| RAPGEF1 | Forward | TTCCACTCACCCAAAATCAA |
|  | Reverse | TTCTACTGCCTGCTGCTCC |
| ATP6V0E1 | Forward | ACGCGGTCAGCTATTGACAC |
|  | Reverse | TAACTCCCCGGTTAGGACCC |
| β-actin | Forward | CTACCTCATGAAGATCCTCACCGA |
|  | Reverse | TTCTCCTTAATGTCACGCACGATT |

**Supplementary Table S2. Nucleotide sequences for transfection**

|  |  | Nucleotide sequences (5’ to 3’) |
| --- | --- | --- |
| si-RPL34-AS1-1 | Forward | GUCAAGGCCACGCAGAAAUCUdTdT |
|  | Reverse | AGAUUUCUGCGUGGCCUUGACdTdT |
| si-RPL34-AS1-2 | Forward | GUCCUGCAUAGCGUGCUUACAdTdT |
|  | Reverse | UGUAAGCACGCUAUGCAGGACdTdT |
| si-RPL34-AS1-3 | Forward | GCACCUGGCUCUAAUAGUUGAdTdT |
|  | Reverse | UCAACUAUUAGAGCCAGGUGCdTdT |
| si-RPL34-AS1-NC | Forward | GUGAGCGUCUAUAUACCAUdTdT |
|  | Reverse | AUGGUAUAUAGACGCUCACdTdT |
| miR-575 mimics | Forward | GAGCCAGUUGGACAGGAGC |
|  | Reverse | GCUCCUGUCCAACUGGCUC |
| miR-NC | Forward | UCACAACCUCCUAGAAAGAGUAGA |
|  | Reverse | UCUACUCUUUCUAGGAGGUUGUGA |
| miR-575 inhibitor |  | GCUCCUGUCCAACUGGCUC |
| miR-inhibitor-NC |  | UCUACUCUUUCUAGGAGGUUGUGA |
| pcDNA3.1-RPL34-AS1 | Forward | CCCAAGCTGGCTAGCGTTTAAACTTAAGCTTGATGCAGGGAACCACCACGTTGTACGGC |
|  | Reverse | GTTTAAACGGGCCCTCTAGACTCGAGCGGCCGCGTGTCTTAAATCCCAGTGTTTATTG |

**Supplementary Fig. S1.** Kaplan-Meier survival curves of ESCC patients with low and high RPL34-AS1 expression.

**
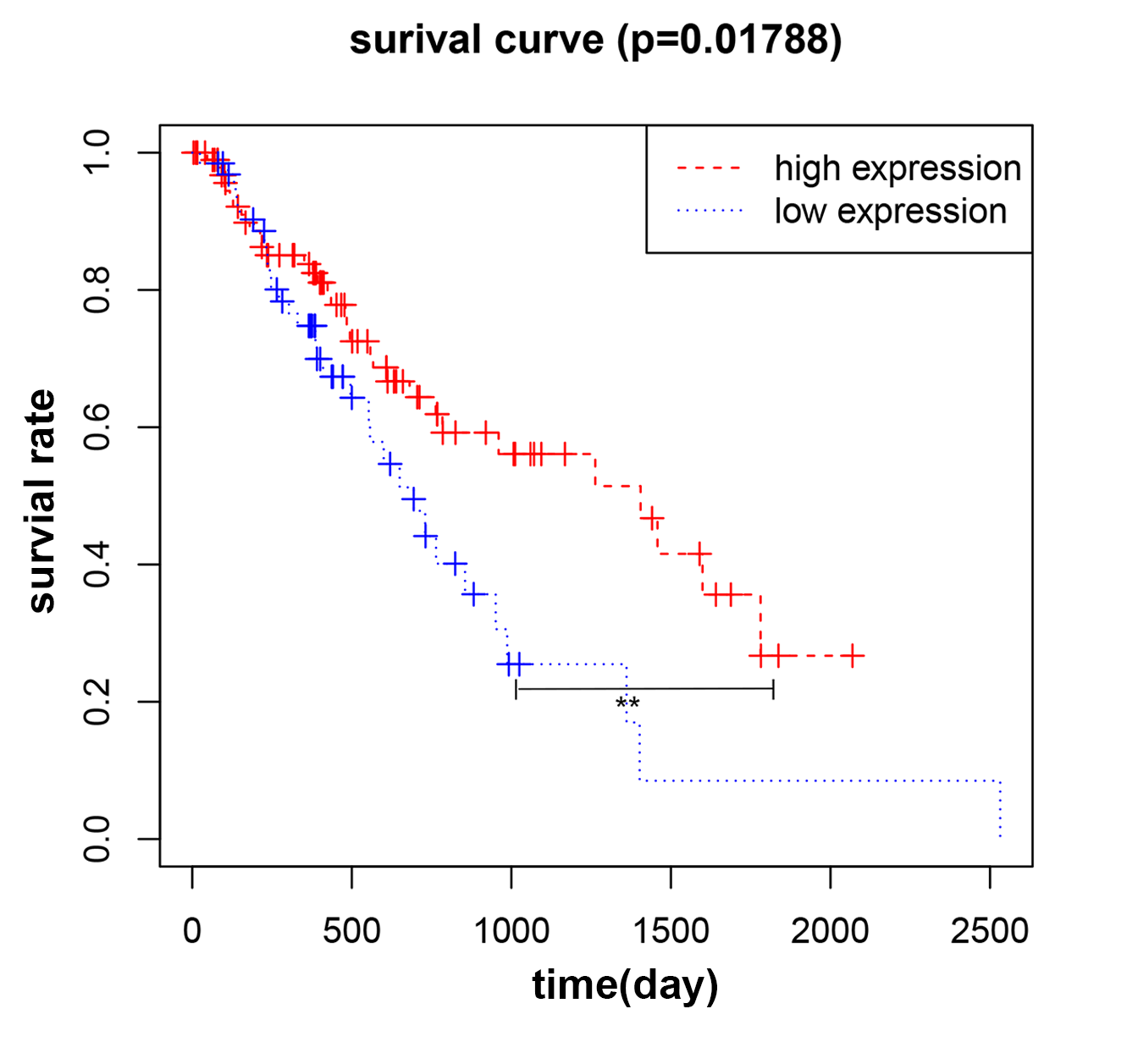
**

**Supplementary Fig. S2.** The lncRPL34-AS1-miRNA-mRNA network diagram was screened by miRanda and TargetScan algorithm.

**
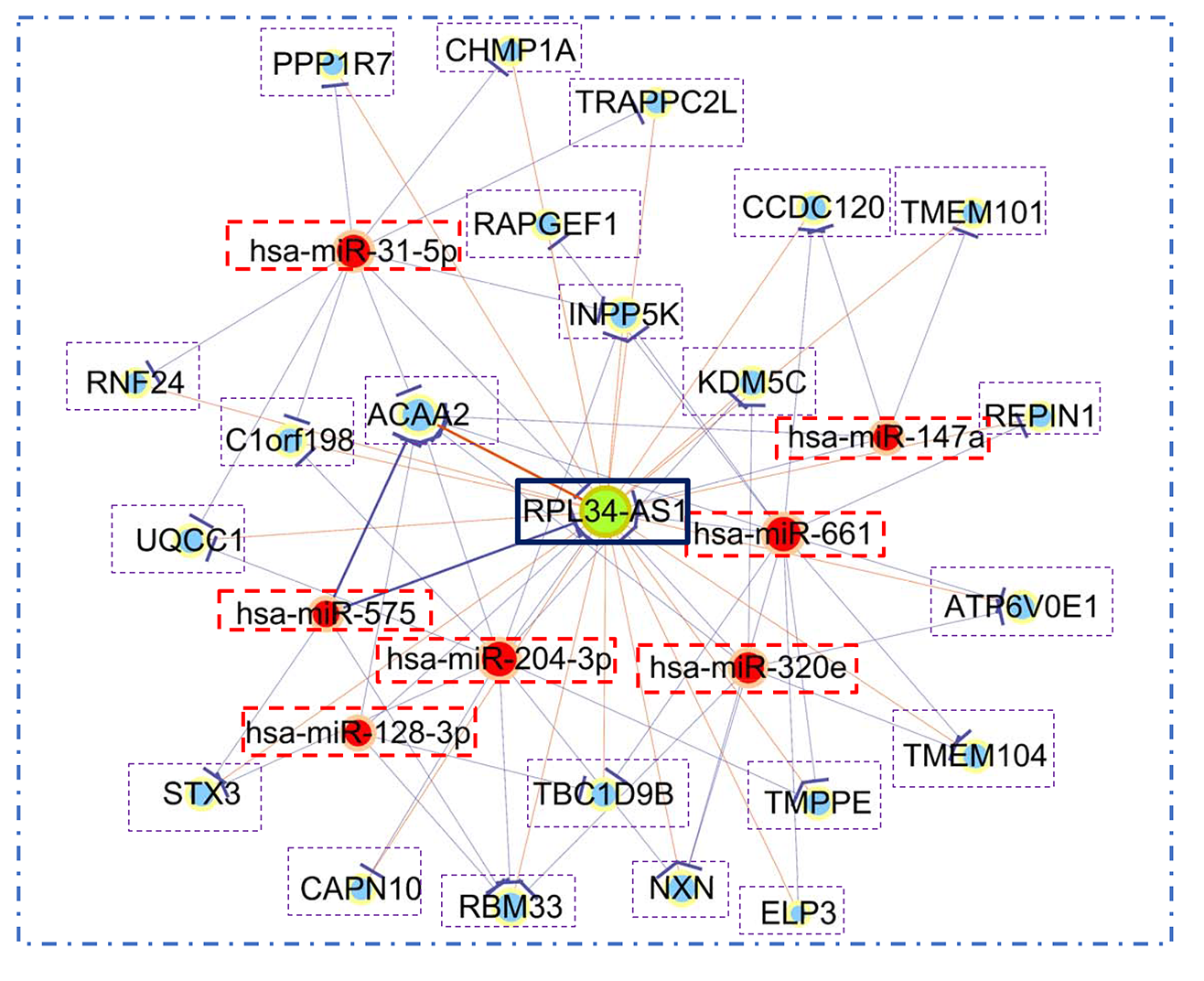
**

**Supplementary Fig. S3.** The RNA-seq results and ceRNA targets to take the intersection in Venny and DAVID bioinformatic analysis.

**
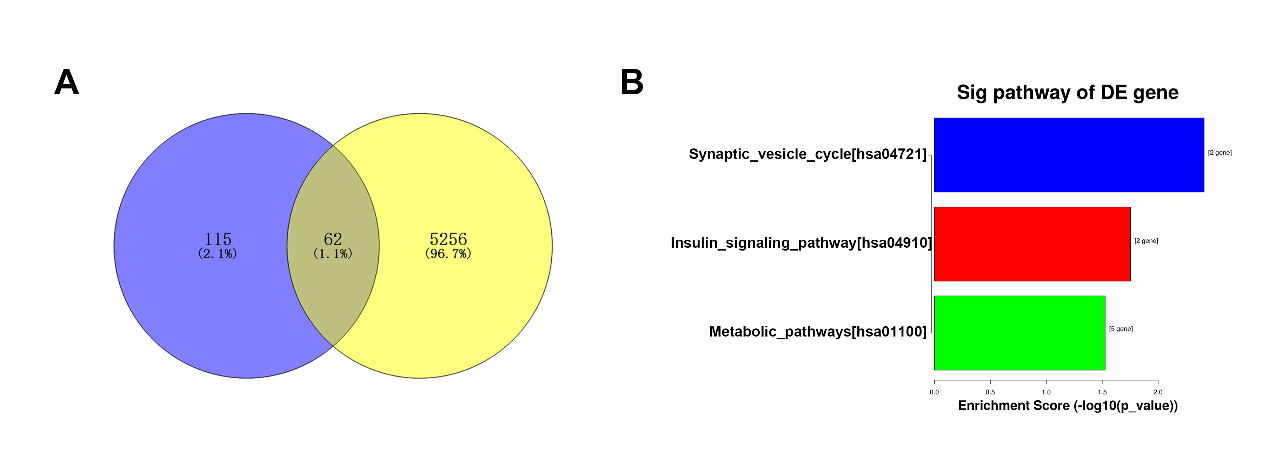
**

**Fig S3. Venny** **and DAVID bioinformatic analysis**. **A**. The intersection mRNA genes of RNA-seq expressed genes and ceRNA targets were displayed in Venny. **B.** The functional pathways were enriched via DAVID bioinformatics resources.

**Supplementary Fig. S4.** The targeted genes expression after knockdown or overexpression of lncRPL34-AS1 in EC9706 cells.


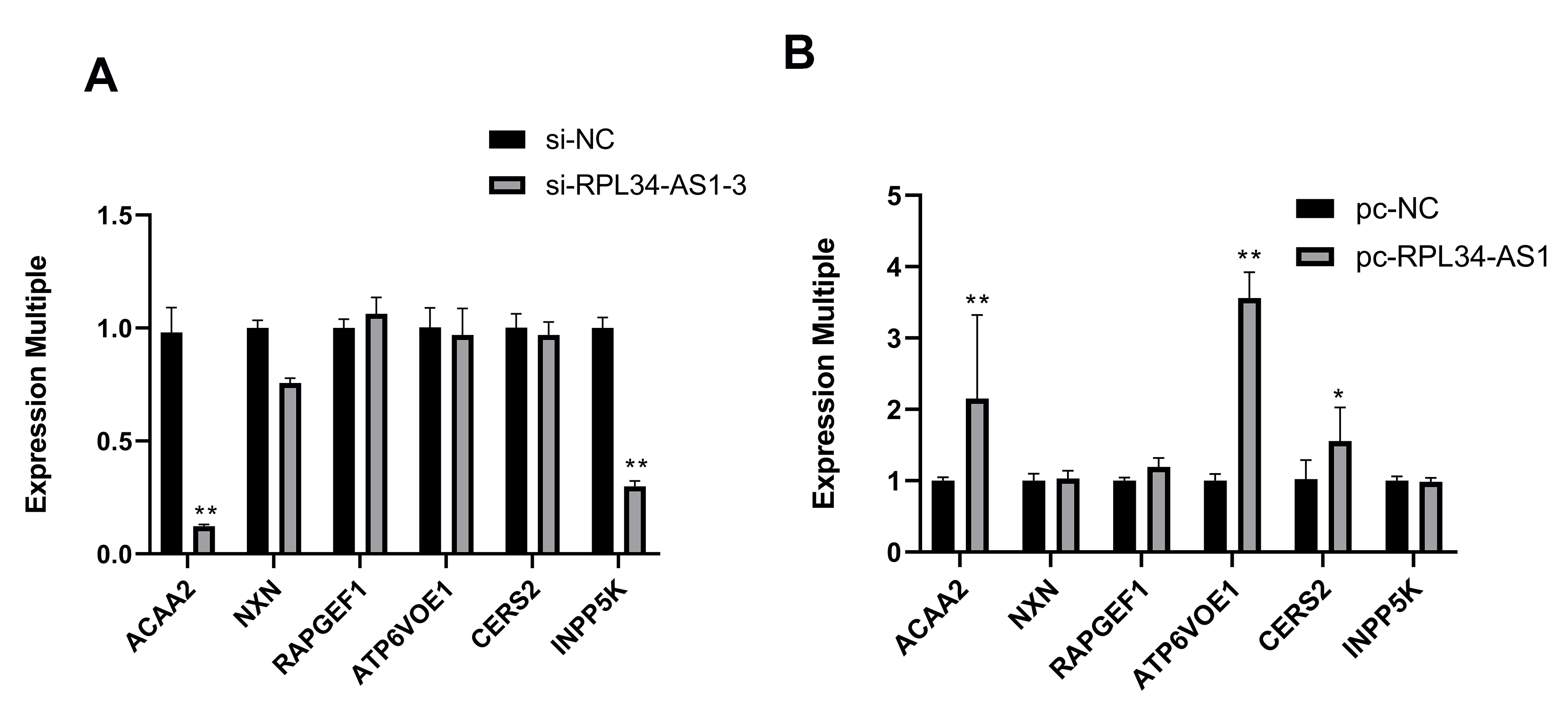


**Fig.S4. The targeted mRNAs expression was detected by RT-qPCR**. **A-B**. Downregulation and of upregulation of RPL34-AS1 in EC9706 cells. Data were showed as mean ± SD. **P* < 0.05, ***P* < 0.01.

**Supplementary Fig. S5.** MiR-575 promoted ESCC cells proliferation, migration and invasion in vitro by targeting ACAA2 in EC9706 cells.

**
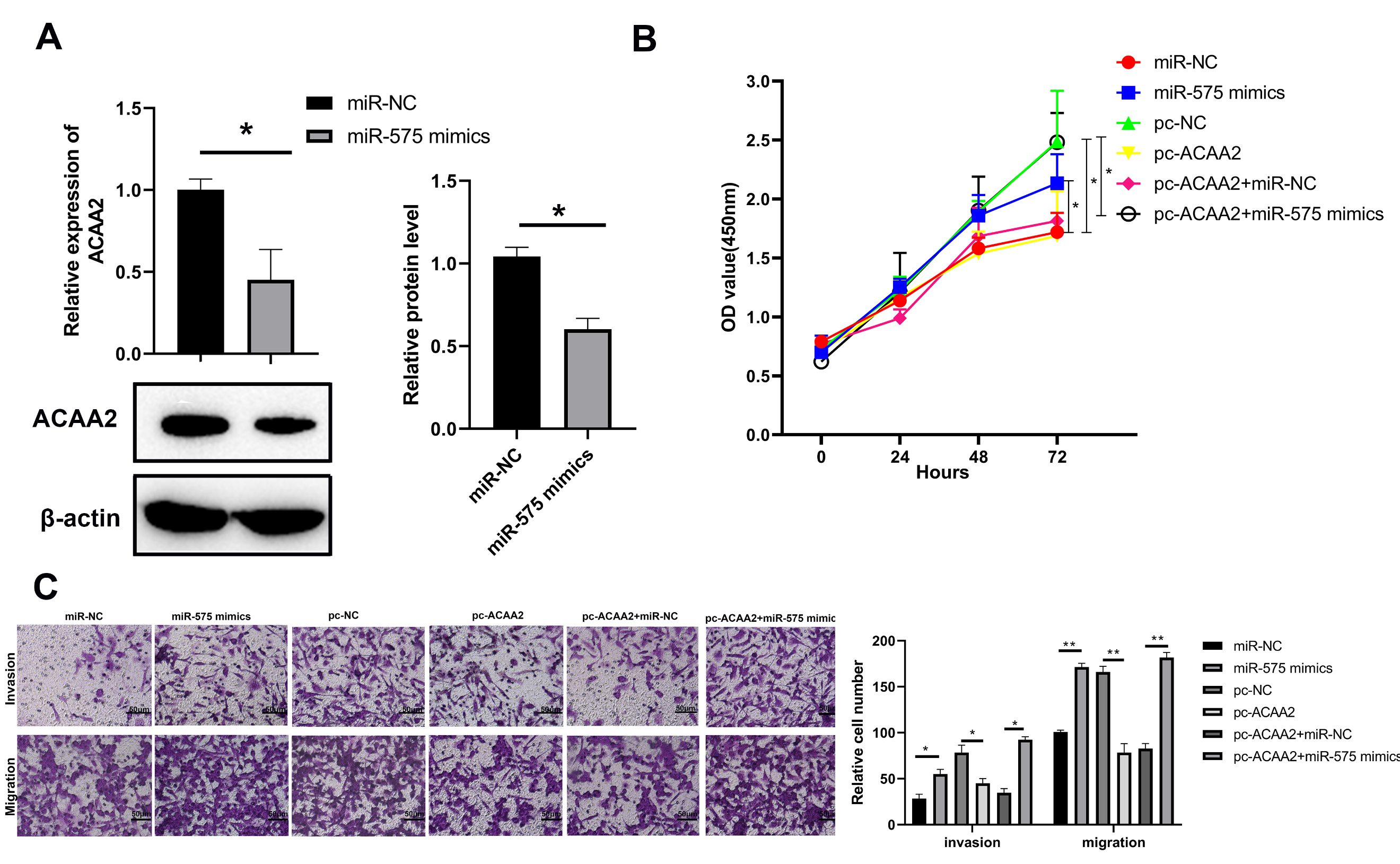
**

**Fig. S5.** MiR-575 acted a promoter of EC9706 cells proliferation, migration and invasion in vitro by targeting ACAA2. **A.** Relative mRNA expression and protein level of ACAA2 were evaluated by RT-qPCR and western blot analysis in EC9706 cells transfected with the miR-575 mimics. **B.** CCK-8 assays were performed to determine the ability of proliferation in EC9706 cells transfected with miR-575 mimics, miR-NC, pc-ACAA2, pc-NC, pcACAA2+miR-NC and pcACAA2+miR-575 mimics. **C.** The cell migratory and invasive capabilities were assessed by transwell assays in EC9706 cells transfected with miR-575 mimics, miR-NC, pc-ACAA2, pc-NC, pcACAA2+miR-NC and pcACAA2+miR-575 mimics. Scale bar, 50 μm. Data were showed as mean ± SD. **P* < 0.05, ***P* < 0.01.

**Supplementary Fig S6.** The original blots of images of Fig. 5A


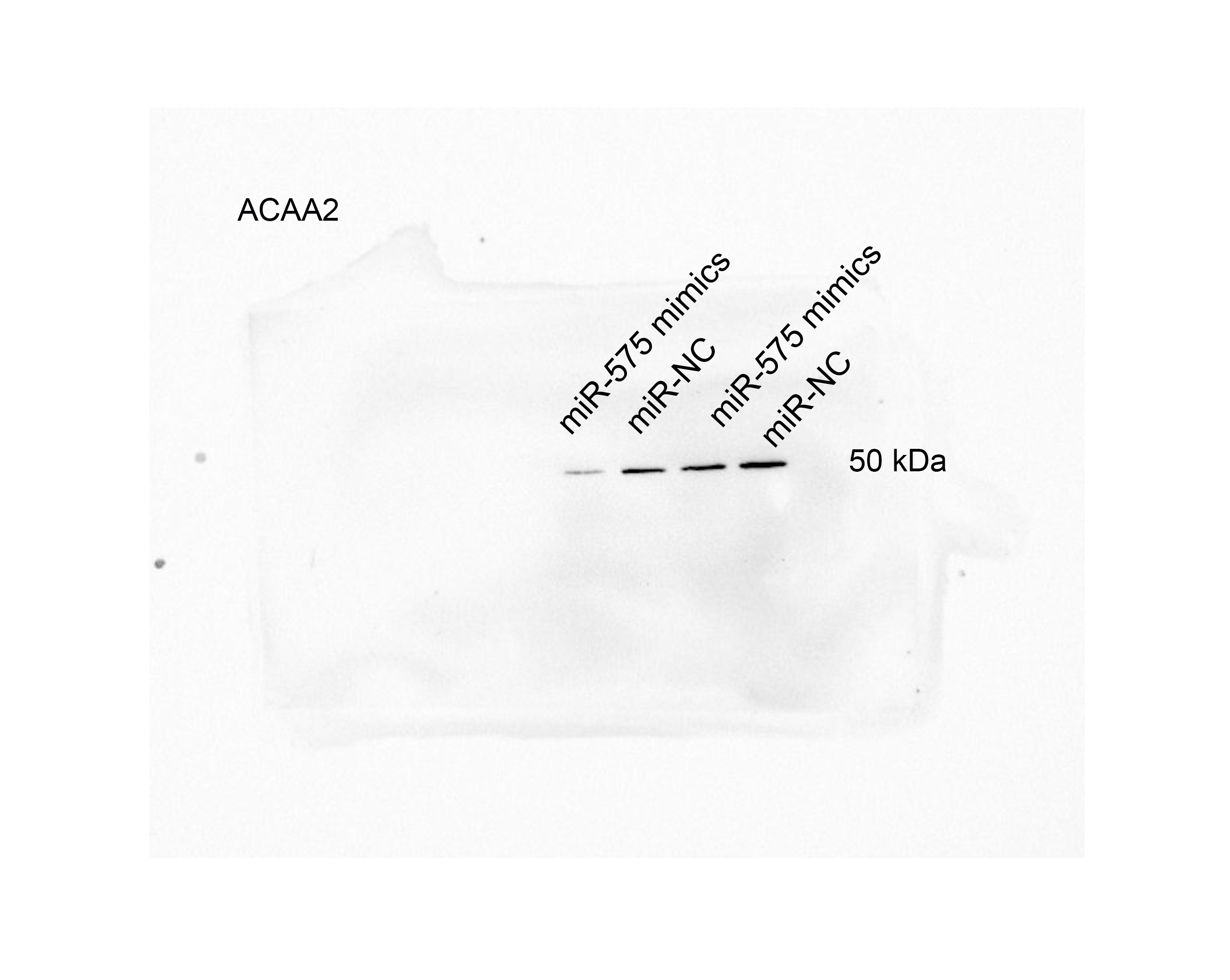

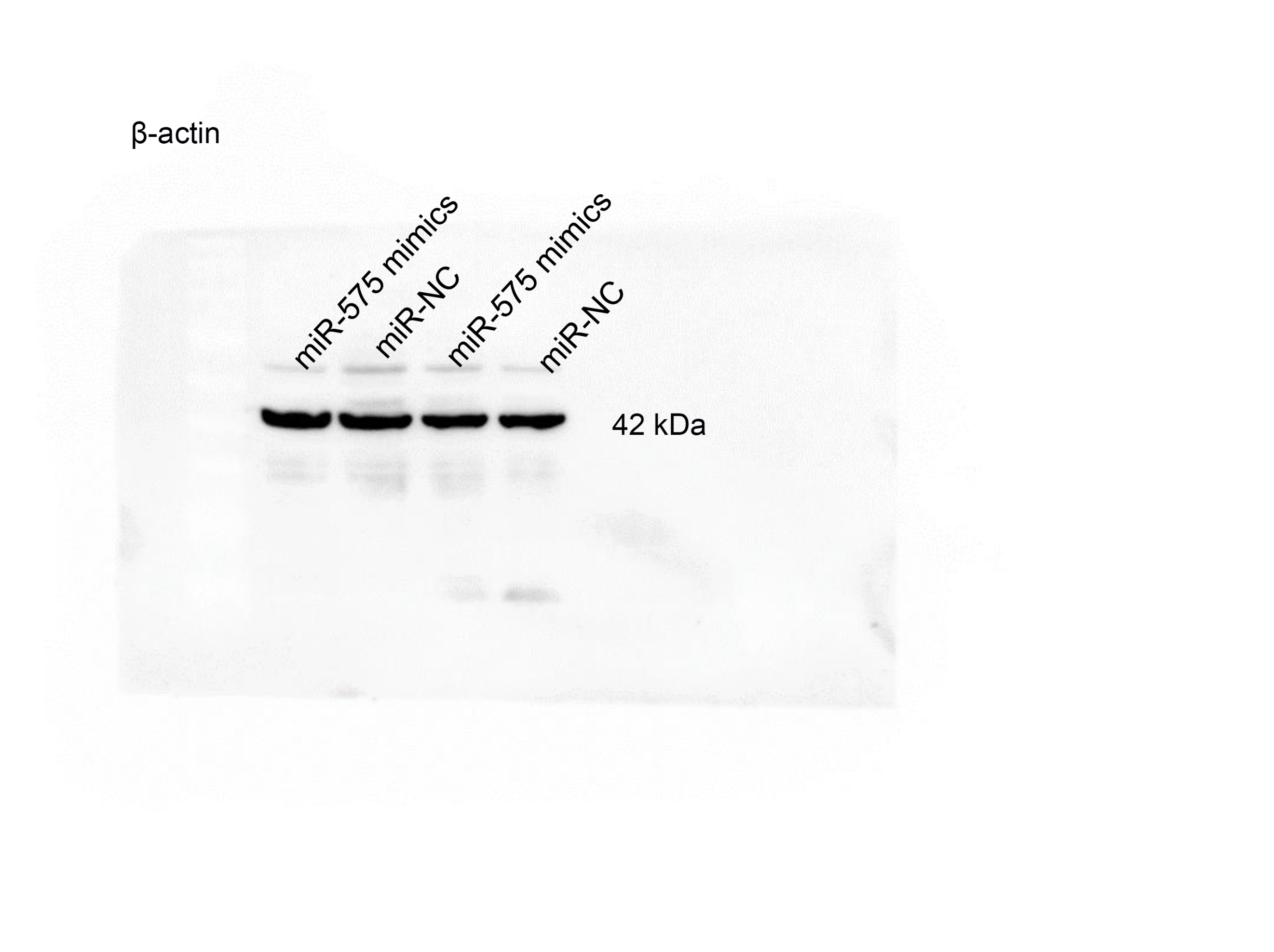


**Supplementary Fig S7.** The original blots of images of Fig. 6A


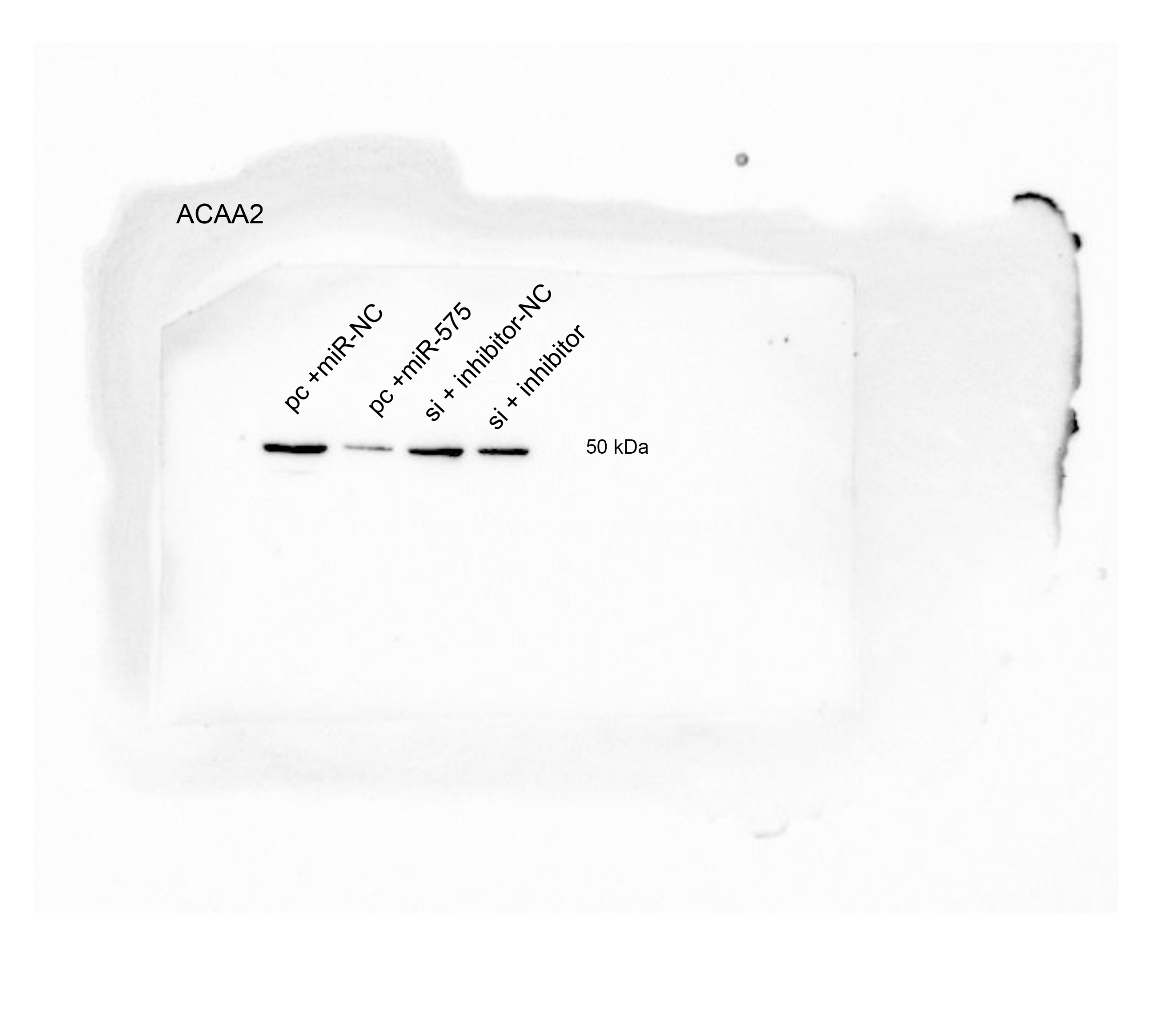

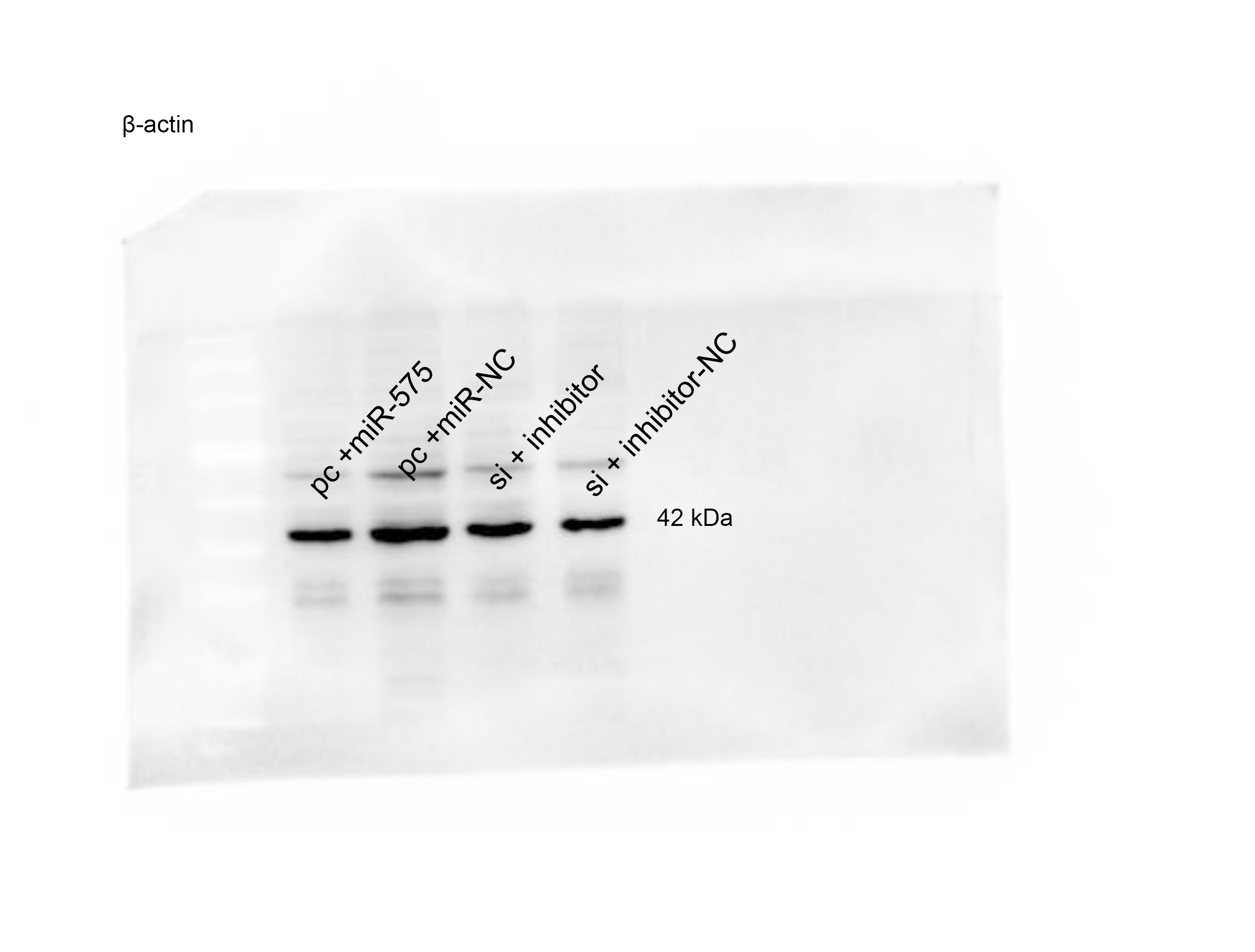


**Supplementary Fig S8.** The original blots of images of Fig. S5A.


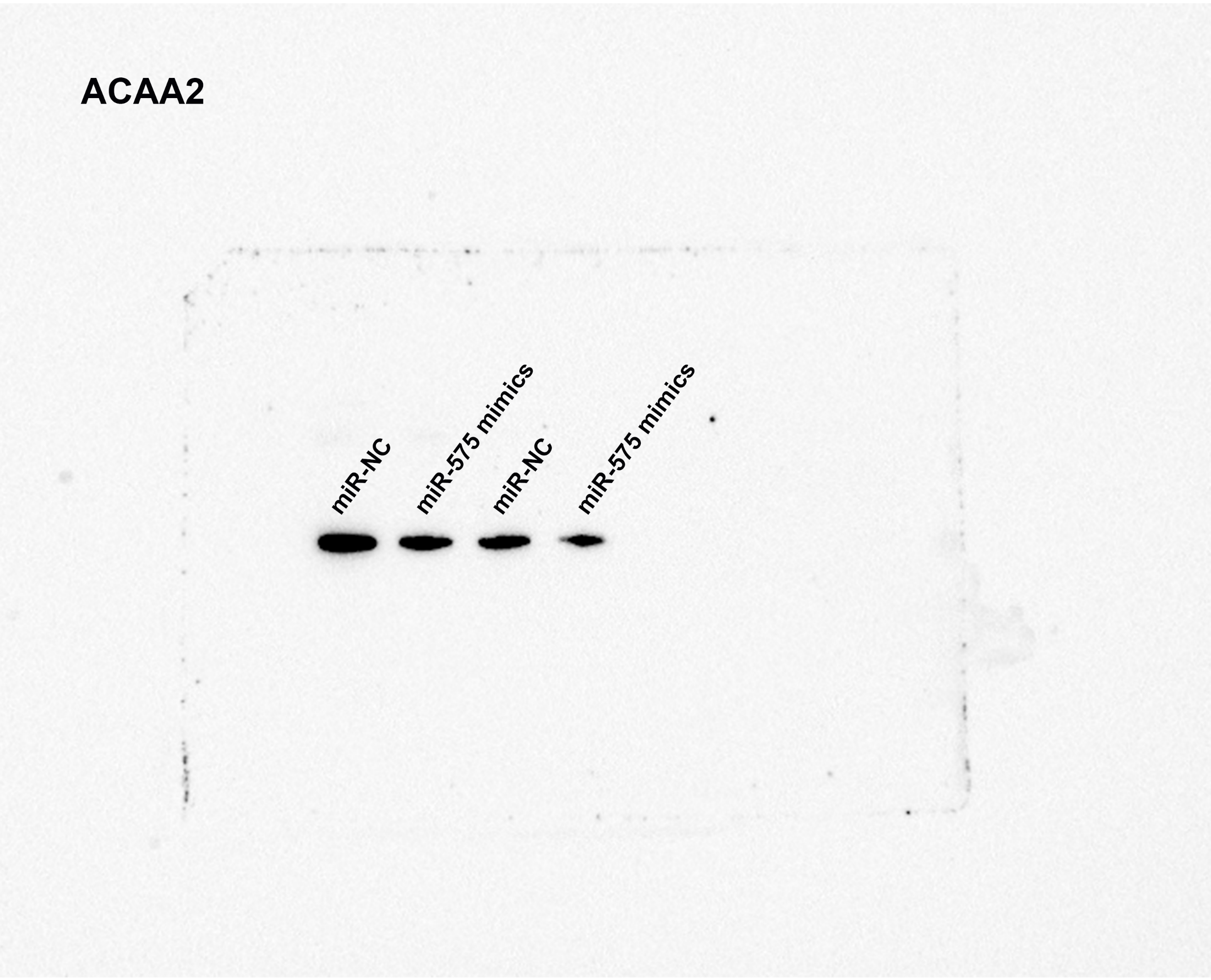


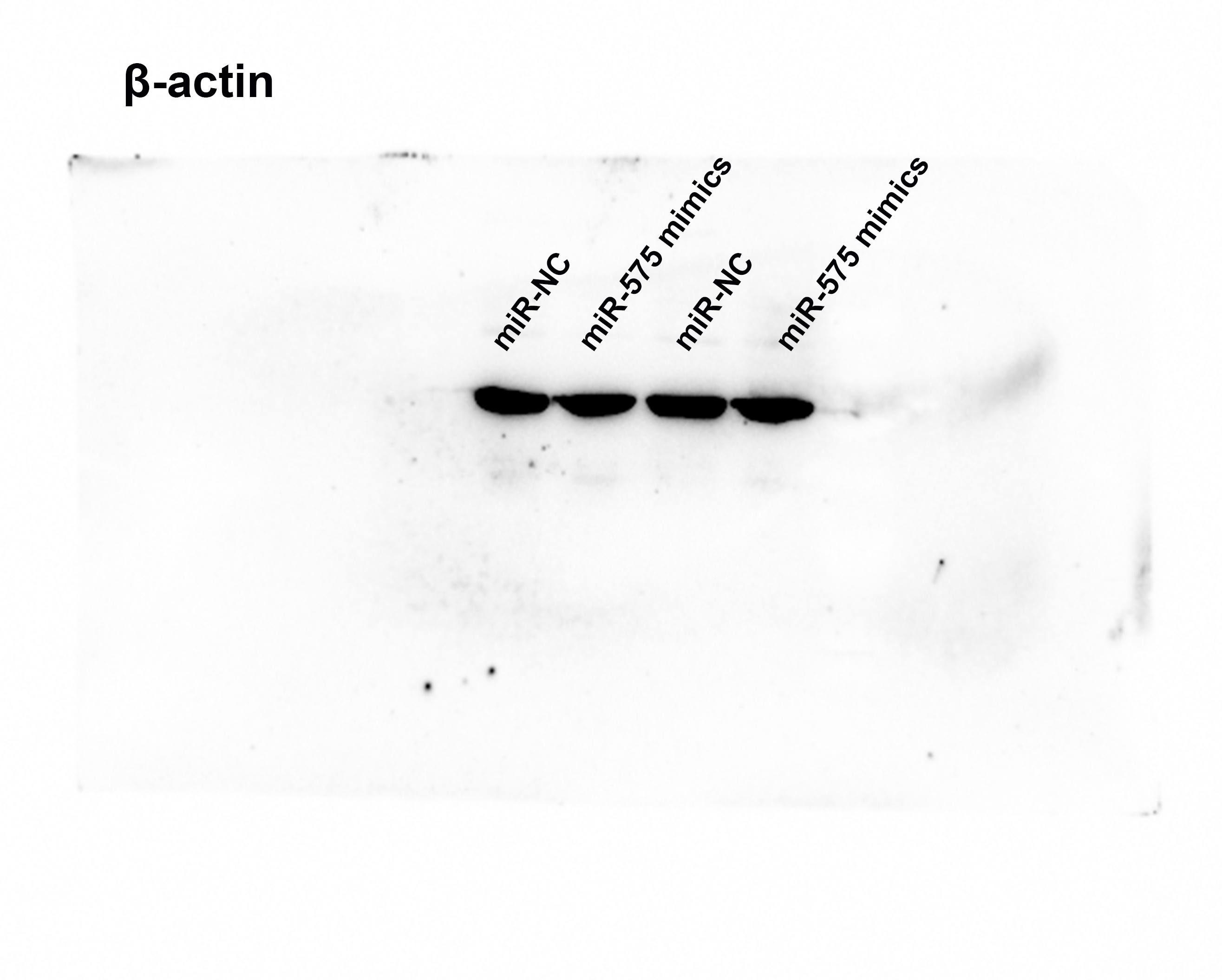

Supplement: Supplementary file 1 — Additional file 1: Supplementary Table S1. Primers for RT-qPCR. Supplementary Table S2. Nucleotide sequences for transfection. Supplementary Fig. S1. Kaplan-Meier survival curves of ESCC patients with low and high RPL34-AS1 expression. Supplementary Fig. S2. The lncRPL34-AS1-miRNA-mRNA network diagram was screened by miRanda and TargetScan algorithm. Supplementary Fig. S3. The RNA-seq results and ceRNA targets to take the intersection in Venny and DAVID bioinformatic analysis. Fig. S3. Venny and DAVID bioinformatic analysis. A. The intersection mRNA genes of RNA-seq expressed genes and ceRNA targets were displayed in Venny. B. The functional pathways were enriched via DAVID bioinformatics resources. Supplementary Fig. S4. The targeted genes expression after knockdown or overexpression of lncRPL34-AS1 in EC9706 cells. Fig. S4. The targeted mRNAs expression was detected by RT-qPCR. A-B. Downregulation and of upregulation of RPL34-AS1 in EC9706 cells. Data were showed as mean ± SD. *P < 0.05, **P < 0.01. Supplementary Fig. S5. MiR-575 promoted ESCC cells proliferation, migration and invasion in vitro by targeting ACAA2 in EC9706 cells. Fig. S5. MiR-575 acted a promoter of EC9706 cells proliferation, migration and invasion in vitro by targeting ACAA2. A. Relative mRNA expression and protein level of ACAA2 were evaluated by RT-qPCR and western blot analysis in EC9706 cells transfected with the miR-575 mimics. B. CCK-8 assays were performed to determine the ability of proliferation in EC9706 cells transfected with miR-575 mimics, miR-NC, pc-ACAA2, pc-NC, pcACAA2 + miR-NC and pcACAA2 + miR-575 mimics. C. The cell migratory and invasive capabilities were assessed by transwell assays in EC9706 cells transfected with miR-575 mimics, miR-NC, pc-ACAA2, pc-NC, pcACAA2 + miR-NC and pcACAA2 + miR-575 mimics. Scale bar, 50 μm. Data were showed as mean ± SD. *P < 0.05, **P < 0.01. Supplementary Fig. S6. The original blots of images of Fig. 5A. Supplementary Fig. S7. The original blo [file 12885_2022_10104_MOESM1_ESM.docx]
